# Structural and functional analysis of protective antibodies targeting the threefold plateau of enterovirus 71

**DOI:** 10.1038/s41467-020-19013-3

**Published:** 2020-10-16

**Authors:** Kuan-Ying A. Huang, Daming Zhou, Elizabeth E. Fry, Abhay Kotecha, Peng-Nien Huang, Shu-Li Yang, Kuo-Chien Tsao, Yhu-Chering Huang, Tzou-Yien Lin, Jingshan Ren, David I. Stuart

**Affiliations:** 1 Division of Pediatric Infectious Diseases, Department of Pediatrics, Chang Gung Memorial Hospital, Taoyuan, Taiwan; 2 Research Center for Emerging Viral Infections, College of Medicine, Chang Gung University, Taoyuan, Taiwan; 3 Division of Structural Biology, The Wellcome Centre for Human Genetics, University of Oxford, Headington, Oxford, OX3 7BN UK; 4 Department of Medical Biotechnology and Laboratory Science, College of Medicine, Chang Gung University, Taoyuan, Taiwan; 5 Department of Laboratory Medicine, Chang Gung Memorial Hospital, Taoyuan, Taiwan; 6 Diamond Light Source Ltd, Harwell Science & Innovation Campus, Didcot, OX11 0DE UK

**Keywords:** Viral infection, Virology, Cryoelectron microscopy, X-ray crystallography

## Abstract

Enterovirus 71 (EV71)-neutralizing antibodies correlate with protection and have potential as therapeutic agents. We isolate and characterize a panel of plasmablast-derived monoclonal antibodies from an infected child whose antibody response focuses on the plateau epitope near the icosahedral 3-fold axes. Eight of a total of 19 antibodies target this epitope and three of these potently neutralize the virus. Representative neutralizing antibodies 38-1-10A and 38-3-11A both confer effective protection against lethal EV71 challenge in hSCARB2-transgenic mice. The cryo-electron microscopy structures of the EV71 virion in complex with Fab fragments of these potent and protective antibodies reveal the details of a conserved epitope formed by residues in the BC and HI loops of VP2 and the BC and HI loops of VP3 spanning the region around the 3-fold axis. Remarkably, the two antibodies interact with the epitope in quite distinct ways. These plateau-binding antibodies provide templates for promising candidate therapeutics.

## Abstract (web summary)

To date, no therapeutic agents against enterovirus 71, the causative agent of hand, foot and mouth disease, exist. Here, using Cryo-EM Huang et al. characterize two plasmablast-derived plateau-binding neutralizing antibodies conferring effective protection against lethal EV71 challenge in vivo.

## Introduction

Enterovirus 71 (EV71) continues to cause disease outbreaks around the world. The 2018 outbreak in Vietnam was associated with over 50,000 clinical cases and at least six deaths^1^ and in the same year in Colorado, USA, 16 children with neurological diseases including meningitis, encephalitis and acute flaccid myelitis were identified^2^. No vaccine is currently available in most endemic regions and the treatment of acute infection is mainly supportive. Antibody-mediated immunity plays a significant role in protection against severe EV71 infections and related mortality in humans^3,4^. Recent conceptual and technological advances in monoclonal antibody development are set to make an enormous impact on the field of infectious diseases, particularly in the context of emerging infectious disease outbreaks. The rapid development and strategic deployment of effective, highly specific preventive and therapeutic interventions may have the potential to alter the course of an epidemic^5,6^.

EV71 is a picornavirus and its capsid contains 60 copies of each of four proteins VP1–VP4, arranged with pseudo T = 3 symmetry^7^. The capsid accommodates surface depressions (“canyons”) around the fivefold vertex of each pentamer, which are the proposed interaction site for two identified cell receptors, P-selectin glycoprotein ligand-1 and heparan sulfate glycosaminoglycan^8–10^. The canyon had been assumed to interact with another receptor, scavenger receptor class B member 2 (SCARB2)^11^, but a recent structural study revealed that SCARB2 binds to EV71 on the southern rim of the canyon, rather than across the canyon^12^. A previous study has shown that the fivefold pentameric vertex and canyon region are epitopes for potent and broadly reactive human neutralizing antibodies^13^.

In contrast to the canyon and fivefold vertex epitopes, the margin of the pentamer is not within or adjacent to known receptor binding sites, but we have shown that natural infection can induce neutralizing antibody responses to this part of the viral capsid (notably the so-called the plateau epitope, located close to the icosahedral threefold axes)^13^. Our data suggest that in some humans the polyclonal antibody response can be focused on a sub-region of the plateau epitope to such an extent that it becomes functionally monoclonal, and can select viruses with point mutations, although this event might be rare^13^. We previously reported two human monoclonal antibodies, 34-1-6D and 16-3-4D, that bind to this threefold plateau epitope and have a genotype-biased neutralizing activity^13^; however, the in vivo role of capsid plateau-binding neutralizing antibodies remains unclear. In addition, the structural basis of how this epitope is important for cellular interactions and virus neutralization, which might guide the development of effective vaccines and immunotherapeutics against EV71, is also lacking.

These issues emphasize the need for a deeper understanding of the human immune response to EV71 viruses. To this end we isolate nineteen EV71-specific monoclonal antibodies from an infected donor who develops a serological response that loses substantial reactivity to EV71 particles harbouring a single residue change (VP3 E81K) within the plateau epitope. The antibodies are tested for binding and neutralization of various EV71 genotypes. 8/19 antibodies reacted to the plateau epitope and three of the eight potently neutralize the virus. The cryo-electron microscopy (cryo-EM) structures of the EV71 virion in complex with two representative plateau-binding neutralizing antibodies reveal that their binding footprints are conserved in the great majority of circulating EV71 strains and these antibodies are therapeutic for lethal challenge in a murine infection. Remarkably, a subset of protective and potent plateau-binding antibodies possesses variable domain sequences 99.5–100% identical to germlines, suggesting that the fast and effective elicitation of such antibodies by immunization is feasible.

## Results

### Characterization of antibodies from the infected donor

Donor C was a 5-year-old patient diagnosed with hand, foot and mouth disease due to EV71 infection based on throat viral isolation and positive reverse transcription-polymerase chain reaction.

**Fig. 1:**
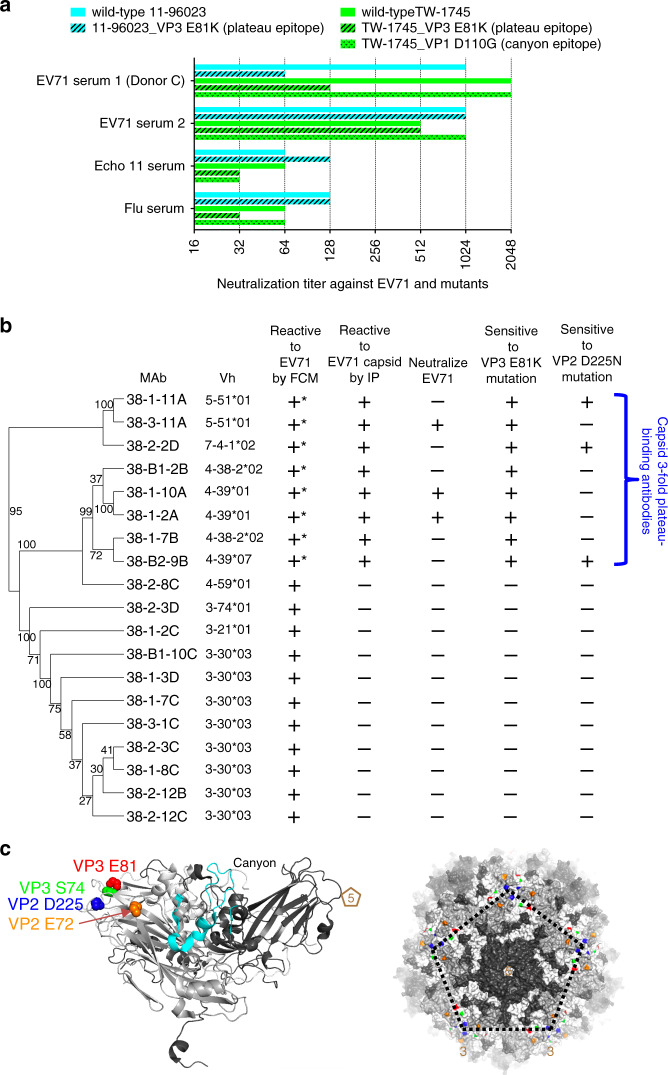
Breadth and specificity of EV71-specific antibody response in donor C. **a** Serological response against wild-type EV71 and its epitope variants. The virus (11-96023_VP3 E81K) was an escape variant selected with neutralizing antibody in vitro^13^ and the TW-1745_VP3 E81K and TW-1745_VP1 D110G were generated in vitro by introducing the residue substitution into an infectious cDNA clone of EV71 (strain TW-1745, GenBank accession number KT354870.1) via site-directed mutagenesis^13^. Donor C developed a strong antibody response at day 9 after illness onset which focused on the threefold plateau epitope of EV71 capsid. A post-EV71 infection serum (day 10 of illness onset) from another paediatric donor, a post-echovirus 11 infection serum (day 10 of illness onset) and a post-influenza virus infection serum (day 11 of illness onset) were included in the experiment. The neutralization assay was carried out twice with equivalent results. Source data are provided as a Source Data file. **b** Characterization of 19 plasmablast-derived EV71-specific monoclonal from donor C, measured by the flow cytometry (FCM), ELISA, immunoprecipitation (IP) and neutralization assays. Those marked by stars bound to purified native viruses in the direct ELISA. Eight of 19 EV71-specific antibodies reacted to conformational epitopes on the viral capsid. A distance tree (cladogram) was constructed by neighbour-joining methods (1000 bootstrap replications) using heavy chain variable domain sequences of 19 EV71-specific human monoclonal antibodies (SeaView software). Bootstrap support values are shown for major nodes on the tree. **c** Mapping of threefold plateau epitope (VP3 E81 coloured in red, VP3 S74 coloured in green, VP2 D225 coloured in blue and VP2 E72 coloured in orange) based on the EV71 capsid structures 4CEY. The side view of a cartoon diagram of EV71 icosahedral asymmetric unit and the surface view of a pentamer are shown with the fivefold vertex at the centre and created using the software program PyMOL. The capsid VP1 protein is coloured in black, VP2 coloured in grey and VP3 coloured in white. The contact region for hSCARB2 cellular receptor^12^ is coloured in cyan.

Donor C’s convalescent serum at day 9 after illness onset showed a strong neutralizing titre between 1:1024 and 1:2048 to wild-type EV71 11-96023 (genotype C4) and TW-1745 (genotype B5) (Fig. 1a). To screen if the antibody response focused on a single antigenic site, the serum was tested against a panel of EV71 escape variants, each of which contained single amino acid mutation within either the fivefold vertex, canyon or plateau epitope^13^ (Supplementary Fig. 1). There was a 16-fold reduction in neutralization titre when serum was reacted with virus harbouring a substitution at VP3 E81K within the plateau, whereas neutralization activity was maintained against other variants. Control sera from echovirus 11 and influenza patients and convalescent serum from another EV71 patient showed minimal or similar activities against wild-type EV71 and its variants (Fig. 1a, Supplementary Fig. 1). The loss of reactivity of donor C’s serum with the VP3 E81K variant suggests that the serum is composed predominantly of antibodies to the threefold plateau epitope at the margin of the pentametric unit^13^.

To explore the neutralization breadth and specificity of the response in donor C, we identified circulating plasmablasts at day 7 after illness onset and sorted single cells to generate human IgG monoclonal antibodies. A total of 42 monoclonal antibodies were produced and 19 of them bound specifically to EV71 in flow cytometry (Fig. 1b, Supplementary Fig. 2). Eight of the nineteen EV71-specific antibodies reacted to purified viruses in enzyme-linked immunosorbent assay and the viral capsid by immunoprecipitation but failed to react to the antigen by immunoblotting, suggesting they recognize a conformational epitope on the viral capsid (Supplementary Fig. 3).

Three (38-1-10A, 38-1-2A and 38-3-11A) of the eight EV71 capsid-reactive antibodies neutralized virus. These neutralizing antibodies were encoded by two diverse heavy chain variable domain (Vh) families, Vh4-39*01 and Vh5-51*01 (Fig. 1b, Supplementary Table 1). Antibody 38-1-2A has identical heavy and light chain variable germline gene segment and CDR3 regions with 38-1-10A, indicating both antibodies were derived from clonally related B cells. Antibodies 38-1-10A and 38-3-11A were chosen as representatives of these families. To define the neutralization epitope, escape mutants of EV71 00-2278 (genotype B4) and 11-96023 (genotype C4) were selected with the two representative antibodies (Supplementary Fig. 4). 38-1-10A selected single mutations VP3 E81K, VP3 S74F or VP2 E72K, each of which was associated with loss of binding and neutralization (Supplementary Fig. 4). Antibody 38-3-11A selected single mutation VP3 E81K, exactly matching one of the 38-1-10A mutations and the previously identified escape mutation for plateau binders, suggesting that these neutralizing antibodies bind overlapping threefold plateau epitopes (Fig. 1c).

The other five of eight EV71 capsid-reactive antibodies did not neutralize and therefore escape mutations could not be selected. However, they all lost binding activities to particles with the single VP3 E81K mutation and two also lost binding activities to the single mutation VP2 D225N variant (Fig. 1b, Supplementary Fig. 2). Antibody 38-1-10A was biotin labelled and a competitive ELISA was performed with these antibodies for binding to the EV71 virion. The result showed that all of them competed to some level with 38-1-10A for binding, and a control threefold plateau-binding antibody (34-1-6D), competed partially with 38-1-10A for binding (Supplementary Fig. 5). In contrast control antibodies including canyon-binding antibody 16-2-12D and twofold plateau-binding antibody 17-1-12A^13^ did not compete with 38-1-10A (Supplemental Fig. 5). These results suggest that all capsid-reactive antibodies from donor C recognize closely related sites on the threefold plateau.

Taken together, eight of eight (100%) of the EV71 capsid-reactive antibodies found in donor C’s antibody repertoire in response to natural infection recognize the threefold plateau epitope, explaining the specificity of his serological response seen in Fig. 1a.

Clonal expansions of EV71-specific plasmablast response were observed within donor C’s antibody repertoires, but each antibody had a unique sequence of rearranged VDJ and VJ segments in the heavy and light chain variable domain when combined with somatic mutations (Supplementary Table 1). EV71-specific antibodies harboured an average of 6 ± 4 and 5 ± 5 non-silent nucleotide mutations in the heavy and light variable (Vl) domain, respectively. There was a clear reduction in the number of variable domain mutations for capsid threefold plateau-binding vs. other antibodies (Vh 2 ± 2 vs. 9 ± 3, *p* = 0.0009; Vl 1 ± 1 vs. 8 ± 4, *p* = 0.0005, Mann–Whitney test) (Supplementary Fig. 6). Plateau-binding neutralizing antibodies were found with no or minimal mutations in the variable domain sequences (Supplementary Table 1) as has also been noted in influenza H7- and hepatitis C virus-neutralizing clones isolated from human donors responding to natural infection or vaccination^14,15^. It is likely that donor C was exposed to EV71 antigen for the first time in their lives during this exposure, and these threefold plateau-binding antibodies may be derived from primary activated B cells through an early round of affinity maturation in the germinal centre.

### Details of threefold plateau-binding neutralizing antibodies

**Fig. 2:**
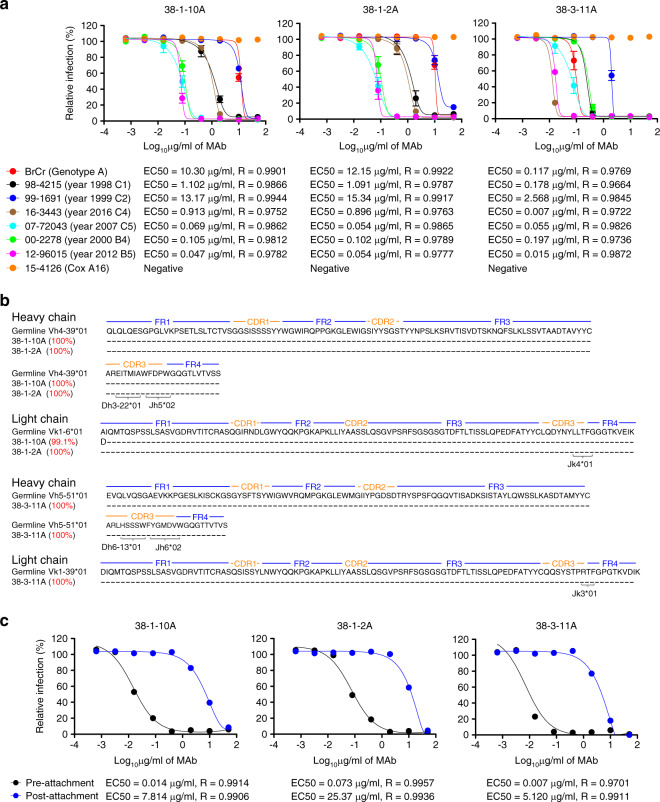
Characterization of threefold plateau-binding neutralizing antibodies. **a** Neutralizing activities of antibodies 38-1-10A, 38-1-2A and 38-3-11A against a panel of EV71 clinical strains in 1998–2016 and the prototype BrCr strain. The neutralization experiment was conducted three times independently (*n* = 3). Data are presented as mean ± standard error of the mean. Error bars represent the standard error of mean. The concentration of antibody that inhibited infection by 50% (EC50) was determined using nonlinear regression (log concentration vs. response, variable slope) in GraphPad Prism. A Coxsackievirus A16 was included in the neutralization assay as the control. **b** Immunogenetic analysis of the heavy and light chain variable regions of neutralizing antibodies using the IMGT tool. **c** Inhibition of EV71 12-96015 infectivity at pre- and post-attachment stages by neutralizing antibodies. Data are presented as the mean for measurements from two independent experiments (*n* = 2). The concentration of antibody that inhibited infection by 50% (EC50) was determined using nonlinear regression (log concentration vs. response, variable slope) in GraphPad Prism. Source data are provided as a Source Data file.

The three neutralizing antibodies, 38-1-10A, 38-1-2A and 38-3-11A, were expanded, purified and their neutralizing activity against a variety of EV71 viruses of genotypes A, B and C characterized in detail. Two previously reported threefold plateau-binding neutralizing antibodies 34-1-6D and 16-3-4D were included in the study (Fig. 2a, Supplementary Fig. 7). 34-1-6D and 16-3-4D originated from two other paediatric donors (16-3-4D from donor M, 34-1-6D from donor Z^13^), using different VDJ rearrangements of Vh domains.

Antibodies 38-1-10A, 38-1-2A and 38-3-11A cross-neutralized EV71 strains of different genotypes. They strongly neutralized EV71 16-3443 (C4), 07-72043 (C5) and 12-96015 (B5) with EC50 values less than 100 ng/ml and 38-3-11A neutralized EV71 of genotype C4 and B5 with EC50 values of 7 and 15 ng/ml, respectively, (Fig. 2a). By contrast, they relatively weakly neutralized EV71 BrCr (A), 98-4215 (C1) and 99-1691 (C2) (Fig. 2a). Antibodies 34-1-6D and 16-3-4D had comparable neutralizing abilities against EV71 00-2278 (B4) and 12-96015 (B5) (EC50 values less than 10 µg/ml); however, both failed to neutralize EV71 98-4215 (C1) and 99-1691 (C2) and 16-3-4D did not neutralize 16-3443 (C4) virus (Supplementary Fig. 7).

We analyzed the sequences of neutralizing antibodies 38-1-10A, 38-1-2A and 38-3-11A using the IMGT tool to determine its closest Vh and Vl germline genes. Interestingly, we found that their Vh genes shared 99.1–100% identity with germlines (IGHV4-39*01 for 38-1-10A and 38-1-2A; IGHV5-51*01 for 38-3-11A) and harboured identical framework and complementarity-determining region genes (Fig. 2b). The antibody Vl genes were also identical to germlines (IGKV1-6*01 for 38-1-10A and 38-1-2A; IGKV1-39*01 for 38-3-11A). The same Vh/Vl pairing, IGHV5-51/IGKV1-39 for 38-3-11A, also exists in the previously described antibodies including the human anti-human immunodeficiency virus antibody m66^16^, West Nile virus antibody CR4285^17^, IgG memory antibody 174-60 from a SLE patient^18^ and germline antibody 5-51/O12^19^, indicating that this is an immunologically relevant cognate Vh/Vl pairing within the human antibody repertoire. 38-1-10A and 38-1-2A proliferated from the same clone. The preferential use of IGHV4-39 in the gene was not only found in 38-1-10A and 38-1-2A (two of the three neutralizing antibodies in donor C) but also in 16-3-4D, which is representative of clonal expansion of plateau-binding neutralizing antibodies (10 of 20 neutralizing antibodies in donor M^13^). Taken together, the abundance and preferential use of these IGHV and IGLV gene families in the antibody repertoire indicates the potential of eliciting 38-3-11A or 38-1-10A-like neutralizing antibodies by vaccination.

To explore the mechanism of neutralization, we performed pre- and post-attachment neutralization assays. Pre-incubation with neutralizing antibodies prevented subsequent viral infectivity of rhabdomyosarcoma (RD) cells in a dose-dependent manner. As shown in Fig. 2c, threefold plateau-binding neutralizing antibodies 34-1-10A, 38-1-2A and 38-3-11A had potent inhibitory activities pre-attachment. In contrast, neutralization at the post-attachment stage required more than a 100-fold greater antibody concentration.

### In vivo protection of threefold plateau-targeting antibodies

**Fig. 3:**
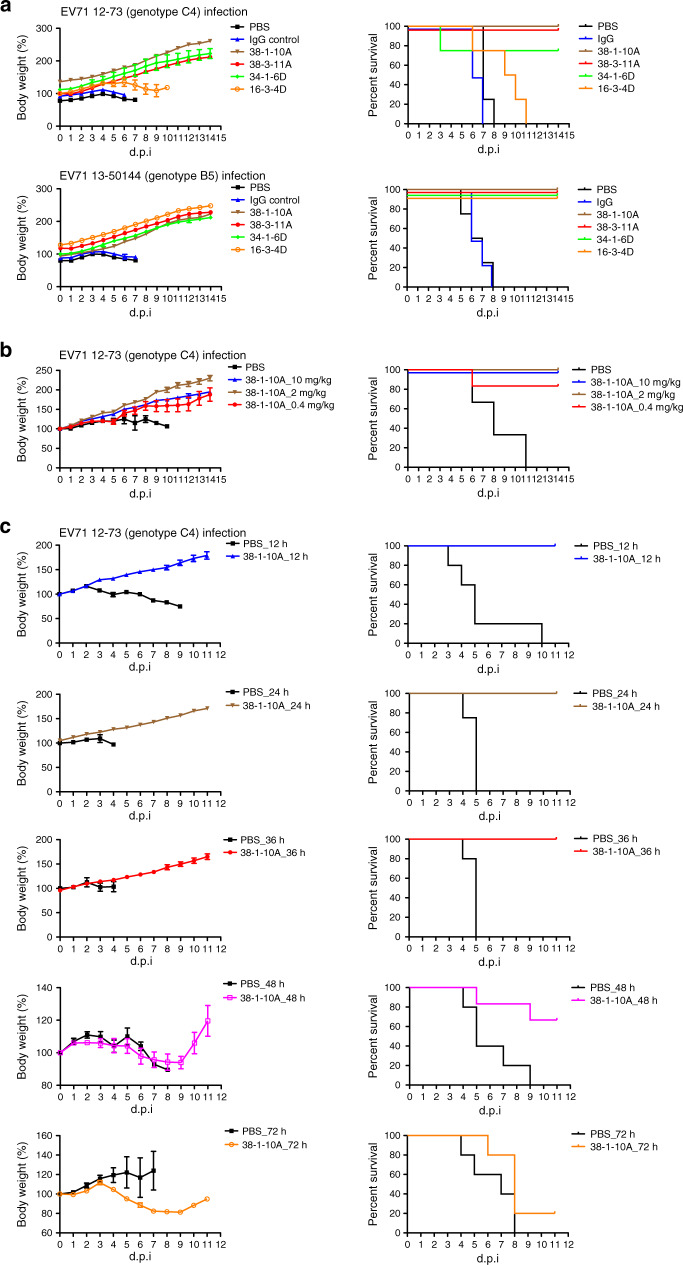
In vivo protection by threefold plateau-binding neutralizing antibodies. **a** The in vivo prophylactic effects of neutralizing antibodies 38-1-10A, 38-3-11A, 34-1-6D and 16-3-4D against EV71 12-73 (genotype C4) (upper) and 13-50144 (genotype B5) (below) infection (PBS control, *n* = 4; IgG control, *n* = 4; 38-1-10A, *n* = 4; 38-3-11A, *n* = 4; 34-1-6D, *n* = 4; 16-3-4D, *n* = 4). A single dose of antibody (10 mg/kg) or control was administered 24 h prior to intraperitoneal infection in the prophylactic experiment. The experiments were performed with PBS and anti-influenza human IgG antibody 4A-14 as controls. **b** The in vivo prophylactic effects of neutralizing antibodies 38-1-10A at doses of 10, 2 and 0.4 mg/kg against EV71 12-73 infection (PBS control, *n* = 6; 38-1-10A 10 mg/kg, *n* = 6; 38-1-10A 2 mg/kg, *n* = 5; 38-1-10A 0.4 mg/kg, *n* = 6). **c** The in vivo therapeutic effects of neutralizing antibody 38-1-10A administered at different timepoints against EV71 12-73 infection (PBS control 12 h, *n* = 5, 38-1-10A 12 h, *n* = 5; PBS control 24 h, *n* = 4, 38-1-10A 24 h, *n* = 5; PBS control 36 h, *n* = 5, 38-1-10A 36 h, *n* = 5; PBS control 48 h, *n* = 5, 38-1-10A 48 h, *n* = 6; PBS control 72 h, *n* = 5, 38-1-10A 72 h, *n* = 5). A single dose of antibody (10 mg/kg) or PBS control was administered 12, 24, 36, 48 and 72 h after intraperitoneal infection in the therapeutic experiment. Body weight was measured at indicated timepoints and data were normalized to the initial weight of each mouse. Error bars represent the standard error of mean. d.p.i. day post infection. Source data are provided as a Source Data file.

To examine the in vivo effect of threefold plateau-targeting neutralizing antibodies, we tested their protective effect in the hSCARB2-transgenic mice model. Mice were treated via the intraperitoneal route with a single dose of 10 mg/kg antibody per mouse 24 h prior to intraperitoneal challenge with a 10 times 50% lethal dose (LD50) of EV71 viruses. Clinical manifestations (weight loss) and mortality in the mice were monitored for 14 days. As shown in Fig. 3a, all mice in the control group (PBS or human IgG control) gradually lost weight and died after challenge. In contrast, neutralizing antibodies 38-1-10A, 38-3-11A and 34-1-6D conferred full protection from lethality by EV71 in the infected mice, while 16-3-4D was only partially protective for the mice.

To explore the prophylactic effect of neutralizing antibody 38-1-10A at different doses, groups of hSCRAB2-transgenic mice were treated intraperitoneally with 10 (high dose), 2 (medium dose) or 0.4 (low dose) mg/kg of antibody 24 h prior to lethal challenge. As shown in Fig. 3b, all three doses completely protected against weight loss, while the high and medium doses completely protected against lethality, with the low dose being partially (80%) protective for the infected mice.

We also tested the therapeutic efficacy of neutralizing antibody 38-1-10A against EV71 in hSCARB2-transgenic mice. Mice were treated with 10 mg/kg of the antibody 12, 24, 36, 48 and 72 h after lethal challenge. Untreated control mice all died after challenge. Figure 3c shows that antibodies given at 12, 24 and 36 h were able to fully protect mice from lethal infection with EV71. Mice treated 48 h after challenge were partially protected (67%), most showing weight loss but then recovering. In contrast, antibody treatment 72 h after challenge only provided a minimal protection from lethality (20%) and substantial weight loss was observed.

### EV71 complexes with 38-1-10A and 38-3-11A Fabs

**Fig. 4:**
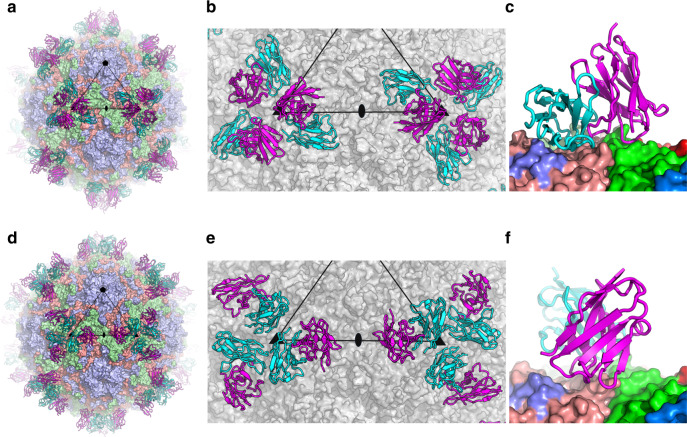
Overall structures of EV71/38-1-10A and EV71/38-3-11A complexes. **a** The structure of the EV71/38-1-10A complex viewed along an icosahedral twofold axis. The viral capsid proteins are shown as surface representations coloured in light blue, pale green and salmon for VP1, VP2 and VP3, respectively. For clarity, only the Vh and Vl domains of the Fab are shown as ribbons with Vh in magenta and Vl in cyan. One icosahedral asymmetric unit of the viron is outlined. **b** Close-up of 38-1-10A Fab binding around two threefolds. The colour scheme is as for a, but the virus is shown in grey. **c** Side view of **b**. Only one Fab is shown for clarity. **d**–**f** EV71/38-3-11A complex. The drawing, colour scheme and view direction of each panel are the same as that of **a**–**c**.

**Fig. 5:**
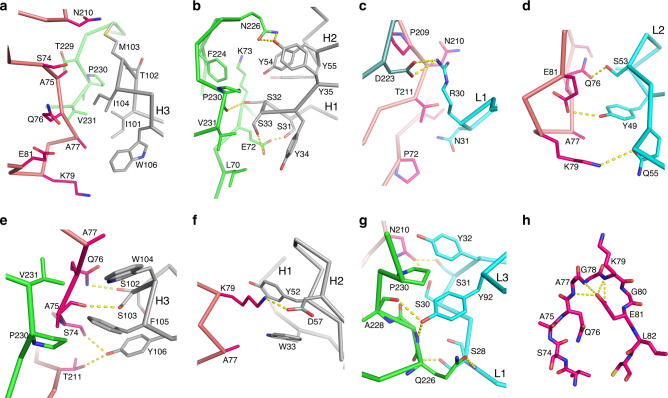
Details of EV71 and Fab interactions. Interactions of EV71 with H3 (**a**), H1 and H2 (**b**), L1 (**c**) and L2 (**d**) loops in the EV71/38-1-10A complex. Interactions of EV71 with H3 (**e**), H1 (**f**) and L1 and L3 (**g**) loops in the EV71/38-3-11A complex. The main-chain backbones are drawn as thick sticks and side chains as thinner sticks with Vh in grey, Vl cyan, VP2 green and VP3 from a neighbouring protomer in salmon. Hydrogen bonds and salt bridges are shown as broken yellow sticks. **h** VP3 BC loop of EV71. The yellow broken sticks represent hydrogen bonds from the key stabilizing residue E81, which is also an escape mutation for both antibodies.

To understand the functional activities and map the epitopes, complexes of virus with the two antibodies were analysed by cryo-EM. Purified EV71 mature virus (genotype B2) was incubated with either 38-1-10A or 38-3-11A Fab with a large excess of Fabs per epitope (“Methods”) at room temperature for 5–20 min, loaded onto an ultrathin carbon grid and immediately vitrified (see “Methods” section for details of sample production and grid preparation). Micrographs of the EV71/38-1-10A complex were collected from a single grid using a Tecnai Polara microscope, while those of the EV71/38-3-11A complex were collected from a single grid using a Titan Krios Microscope (see “Methods” section). Three-dimensional reconstructions with icosahedral symmetry from 10074 particles of the EV71/38-1-10A complex and 14430 particles of the EV71/38-3-11A complex yielded density maps to 2.7 and 2.8 Å resolution, respectively, (Supplementary Fig. 8). Both Fabs bind close to the threefold icosahedral axis of the virus with a stoichiometry of 60 Fabs per virus particle (Fig. 4). The density for the Fab in the EV71/38-1-10A complex is weaker and has lower resolution than that for the viral capsid, suggesting that not all of the binding sites are occupied. However, most of the side chain orientations of the Fab at the interface with the virus are defined by the density at a lower contour level (Supplementary Fig. 9). To enable the model of the Fab and its interactions with the virus to be accurately modelled, we determined the crystal structure of the 38-1-10A Fab. There were nine 38-1-10A Fabs in the crystal asymmetric unit and the structure was refined to 2.7 Å resolution with good R-factors and stereochemistry (Supplementary Table 2 and Supplementary Fig. 10). The nine copies in the asymmetric unit were essentially identical (rmsd 0.7 Å). The VhVl domains of the crystal structure fit well with the EM map with minor adjustments of some surface loops and side chains. The crystal structure of EV71 (PDB ID 3VBS)^7^ also fitted well into the EM map of both complexes. Based on the B-factors after refinement and the strength of the density, the occupancy of the Fab in the EV71/38-1-10A complex was estimated to be approximately 40%. In contrast the Fab variable domain density for the EV71/38-3-11A complex was well defined and enabled the model to be built without difficulty (Supplementary Fig. 9). In the EM maps of both complexes, the resolution for the constant domains of the Fabs was lower (Supplementary Fig. 8) and only the overall positions and orientations of these domains could be determined. The final refined models for both complexes, each containing the viral capsid proteins VP1-4 and VhVl domains of the Fabs, fit the maps well (Supplementary Table 3) allowing the detailed interactions of a major HFMD causing virus with human neutralizing antibodies to be described for the first time at atomic resolution (Fig. 5).

### Fabs bind overlapping sites with radically different poses

**Fig. 6:**
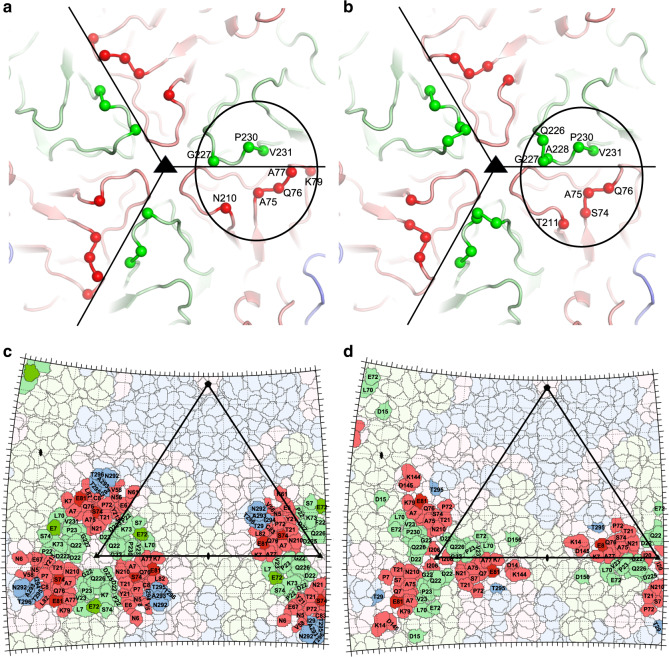
38-1-10A and 38-3-11A interact with the same epitope of EV71. The core region of the epitope that interacts with 38-1-10A (**a**) and with 38-3-11A (**b**). Capsid proteins of EV71 are shown as ribbons with VP1, VP2 and VP3 coloured in light blue, pale green and salmon, respectively. C_αs_ of viral residues that have direct contact with CDR H3 or L3 (≤4.0 Å) are drawn as bright spheres. The core region of one epitope is circled and black lines mark the pentamer boundaries. **c**, **d** Roadmaps of the virus surface with colour scheme as in **a** and an icosahedral subunit outlined in black lines. Residues in contact with 38-1-10A (**c**) or 38-3-11A (**d**) are highlighted in the corresponding viral protein colour and escape mutations are shown in bright colours.

As will be seen below the two antibodies are strikingly similar to each other in structure and 20 residues of the epitope are common between the two antibodies (out of 25 for 38-3-11A and 41 for 38-1-10A, Fig. 6c, d and Supplementary Figs. 11 and 12). Furthermore, as described above, a single escape mutation can abolish the binding of both antibodies. However, the overall modes of engagement with the virus are radically different for the two antibodies and there is not a single interaction pair common between the two. In line with the broad cross reactivity observed, the epitope residues defined from the complexes are almost completely conserved across four genotypes of EV71 (Supplementary Fig. 11).

### Engagement of 38-1-10A Fab with EV71

38-1-10A binds close to the virus icosahedral threefold axis with the heavy and light chains positioned roughly equidistant from the axis, shielding an unusually large area (1400 Å^2^) of virus surface (640 Å^2^ shielded by heavy chain and 760 Å^2^ by light chain). The Vh domain mainly contacts VP2 (footprint 470 Å^2^). The heavy chain CDR H3 (H3) sits above the pentamer boundary and makes predominately hydrophobic interactions with the VP2 HI loop, residues 75–77 of the BC loop and N210 of the VP3 HI loop of a neighbouring protomer (Figs. 4–6). The heavy chain CDR-H1 (H1), which includes a run of serine residues, contacts the BC and HI loop of VP2. There are four potential hydrogen bonds from the carbonyl oxygen of S30 and the hydroxyl groups of S31, S32 and S33 to the hydroxyl group of S74, the side chain of E72, the carbonyl oxygen of P230 and the side chain of E72, respectively, (several shown in Fig. 5). The Vl domain leans on the virus with its β-strands almost parallel to the viral surface such that only the edge of the antigen binding region interacts with the virus. Vl contacts make a large footprint on the protomer adjacent to that interacting with the Vh domain (Figs. 4–6). Contacts are mainly made by CDR-L1 (L1), βC″ strand, βD strand and CDR-L2 (L2) to the VP3 BC loop and HI loop and the VP2 HI loop (Fig. 5). In addition L3 also contacts the VP2 HI loop.

### Engagement of 38-3-11A Fab with EV71

The variable domains of 38-3-11A and 38-1-10A are very similar (rmsd 1.0 Å for 217 of 226 C_α_s). The major differences are in the heavy chain CDR loops. In 38-3-11A H1 is two residues shorter, H2 one residue longer and H3 two residues longer than in 38-1-10A (Supplementary Fig. 12). 38-3-11A binds along the pentamer boundary with the light chain close to the threefold and directed more upwards from the virus surface compared to 38-1-10A, so that the antibody shields a much smaller area of the virus surface (770 Å^2^; 440 Å^2^ by heavy chain and 330 Å^2^ by light chain) (Fig. 4). If we define the viral residues that interact with H3 and L3 of the Fab as the epitope core, it is interesting to note that although 38-3-11A rotates 91° relative to 38-1-10A, their centres of mass overlap such that the H3 and L3 CDRs of both Fabs interact with the same core region of the epitope. However, the 38-3-11A Vh domain footprint is mainly on VP3 of the neighbouring protomer, instead of VP2 for 38-1-10A (Figs. 4–6). H3 of 38-3-11A packs against the BC and HI loops of VP3 from the neighbouring protomer, forming three hydrogen bonds. The aromatic residues at the tip of H3 make hydrophobic interactions with main-chain peptides and alanine side chains of VP3 of the neighbouring protomer, as well as hydrophobic residues of the VP2 HI loop (Fig. 5e). H1 and CDR-H2 (H2) make hydrophobic and salt-bridge interactions with the VP3 BC loop, (Fig. 5f). Y93 of L3 makes substantial hydrophobic, ring stacking and hydrogen bond interactions with the VP2 HI loop, whilst L1 interacts with the HI loops of both VP2 and VP3 via hydrophobic and hydrogen bond interactions (Fig. 5g).

### Structural mechanism of escape

The VP3 BC loop harbours the resistance mutation VP3 E81K, which is selected by both antibodies. Unusually the mutation is not part of the footprint of the antibodies, instead there is likely an indirect effect. The residues preceding E81 loop around it, such that each of the amide groups of residues 77–79 form hydrogen bonds to the negatively charged carboxylate group of residue 81. The outer surface of this loop makes direct interactions with the H3 loops in both complexes (Fig. 5). The E81K substitution would not only destroy the hydrogen bonds inevitably inducing structural changes, but the marked change in electrostatic properties may also contribute to changes in the BC loop structure, disrupting the binding of both Fabs. Mutations S74F in VP3 and E72K in VP2 are also selected by 38-1-10A. S74 lies at the N-terminus of the VP3 BC loop and has direct contacts with N31 of L1 in the EV71/38-1-10A complex and is close to the tip of H3, whilst E72 interacts with H1 making hydrogen bonds to S31 and S33 (Fig. 5). These two mutations are unlikely to alter the local structure of the virus, rather they would directly interfere with Fab binding. Surprisingly VP3 S74 also interacts with the antibody in the EV71/38-3-11A complex, making a hydrogen bond to the hydroxyl group of H3 Y106, however since it is located at the edge of viral-Fab interface (Fig. 5e) the mutation can presumably be accommodated without abolishing binding. In contrast VP2 E72 is not involved in interactions with 38-3-11A, so it is unsurprising that it is not selected as an escape mutation. Finally, D225, which affects the binding of some other antibodies, but not 38-1-10A and 38-3-11A is, as expected, located outside their Fab footprint.

## Discussion

Natural EV71 infection induces a rapid antibody response to the virus capsid and the breadth and potency of the human antibodies elicited correlates with the neutralization epitope they target. This observation emphasizes the antigenic variety of epitopes in the EV71 capsid and warrants investigation of the human antibody repertoire.

We have characterized a panel of EV71 capsid-reactive human monoclonal antibodies from a young child who was naturally infected with EV71, and show that a significant portion (8 of 19, 43%) of plasmablast-derived antibodies targeted the plateau region close to the icosahedral threefold axes, compatible with the total breadth of the serological response. We have also examined two plateau-binding neutralizing antibodies (34-1-6D and 16-3-4D) from other infected donors^13^. From the structural and functional data presented here, we draw the following three conclusions. First, antibodies directed against the plateau epitope, with conserved regions around the interface between VP3 and VP2 proteins, have a variety of potential germline origins (Vh4-38-2, Vh4-39, Vh5-51, Vh7-4-1). Vk1-39 is commonly used among the plateau-binding antibodies we have characterized (Supplementary Table 1). Antibody 38-1-2A has identical heavy and light chain variable germline gene segments and CDR3 regions with 38-1-10A, indicating both antibodies were derived from clonally related B cells. Nevertheless, each of four representative neutralizing antibodies has a unique gene usage of heavy chain VDJ rearrangement and light chain VJ rearrangement.

Secondly, potent and protective plateau-binding antibodies can be rapidly induced by natural infection in young children. Antibodies 38-1-10A and 38-3-11A neutralized virus at picomolar level and fully protected mice from a lethal EV71 infection at a prophylactic dose of 0.4 mg/kg, in accordance with previous results for murine anti-EV71 antibody^20–22^ and similar to the efficacy of monoclonal antibodies against Coxsackievirus A10 and influenza virus^23,24^. Nevertheless, the potency of plateau-binding neutralizing antibodies varies dramatically even when they bind overlapping epitopes. Thus antibody 16-3-4D is less effective against strain 16-3443 (genotype C4) in vitro and 12-73 (genotype C4) in vivo. In the present study plateau-binding neutralizing antibodies were tested in vitro with a small panel of EV71 strains representative of genotype A, C1, C2, C4, B4 and B5 and the relationship of binding affinity and potency by each antibody warrants further investigation.

Thirdly, germline genes are commonly found in the variable domains of EV71 capsid-reactive antibodies. Our results show that antibodies to the capsid threefold plateau epitope, with little or no affinity maturation (0 amino acid mutation in Vh and 0-1 amino acid mutation in Vl for neutralizing antibodies 38-1-10A, 38-1-2A and 38-3-11A; Supplementary Fig. 6), can be induced by natural infection in a naïve donor. Low levels of somatic mutation for similar antibodies isolated after influenza H7 and Ebola exposure^15,25,26^ imply that these antibodies are part of a primary response. The mechanism by which this preference for the germline-encoded sequence in the neutralizing antibody repertoire occurs in our donors is unclear, but possibilities include (i) a skewed primary immune response in the first exposure to a new virus (ii) plasticity of epitope–paratope interactions in germline antibodies that may enhance promiscuity in the primary antibody response^27–29^ and (iii) selection of B cells with high affinity to intrinsically attractive epitopes^30,31^. These mechanisms are not mutually exclusive and may act together.

Our structural results show that both 38-1-10A and 38-3-11A recognize a threefold proximal epitope composed of VP2 BC and HI loops from one protomer and VP3 BC and HI loops of another protomer from a neighbouring pentamer, although the two antibodies are bound in radically different orientations. Such radically different engagement of an epitope has been reported before, for instance for SARS-CoV-2^32^, and indeed in another enterovirus (type 1 poliovirus) plateau antibodies were found to bind two overlapping epitopes^33^. Previously, several lower resolution structural studies of murine EV71-neutralizing antibodies have been reported including E18, E19, D5, A9 and D6^22,34,35^. A9, D6 and E19 recognize an epitope which includes the VP3 knob, VP3 BC loop and VP1 C terminus region from a single protomer^34,35^, whereas D5 recognizes an epitope centred on the VP1 GH loop from one protomer and VP3 GH loop from a neighbouring protomer within the same pentamer^22^. The footprint of E18 is somewhat similar to that of 38-1-10A^34^. More than 20 enteroviruses of types A and B, have been reported to cause HFMD. Type A enteroviruses use either SCARB2 or KREMEN1 as entry receptors^36–38^, and structures of receptor complexes for both have been determined recently^12,39^. SCARB2 binds EV71 on the south rim of the canyon, whilst KREMEN1 engages across the canyon. Binding of either receptor can initiate a cascade of conformational changes that produces expanded A-particles^40,41^. Whilst the footprints of previously reported antibodies have little or no overlap with that of SCARB2, the attached antibodies would clash with the bound SCARB2 receptor. Interestingly, binding of E18 or D6 can also induce strain in the viral capsid and premature release of viral genome^34,35^, mimicking the binding of SCARB2, although how the antibodies trigger conformational changes is not clear. In contrast neither 38-1-10A nor 38-3-11A show any tendency to convert virus to empty capsids or A-particles. Overlaying EV71/38-1-10A and EV71/38-3-11A with EV71/SCARB2 shows severe clashes between the Fabs and receptor, suggesting that neutralization is by preventing receptor binding rather than inducing premature uncoating (Supplementary Fig. 13). This is in line with our observation that all threefold plateau-binding neutralizing antibodies were effective at both the pre- and post-attachment stages, although the concentration required for post-attachment inhibition was much higher than that for pre-attachment inhibition. Nevertheless, potential interference by 38-1-10A or 38-3-11A antibody with the interaction of virus to other cellular receptors (i.e. P-selectin glycoprotein ligand-1 and heparan sulfate glycosaminoglycan)^8–10^ remains unclear.

The prophylactic and therapeutic efficacies of plateau-binding neutralizing antibodies were assessed in hSCARB2-transgenic mice. The results of prophylactic experiments indicated that potent monoclonal antibodies might be promising candidates as a prophylactic for high-risk population (i.e. young infants or immunocompromised individuals) exposed to virus^6,20,26^. The therapeutic experiments showed that 38-1-10A substantially reduced the mortality and loss of body weight when administered within 48 h of challenge. These proof-of-concept experiments are supportive of the use of monoclonal antibody for the post-exposure treatment of lethal EV71 infection, although the mechanism of time dependent effect of 38-1-10A on protection is not well understood. A multi-antibody cocktail approach targeting non-overlapping neutralizing epitopes might improve or enhance the protection^6^. The formulation and timepoint of antibody administration require more investigation to provide a definitive therapeutic strategy in future clinical studies.

In summary we have shown that humans naturally infected with EV71 make an antibody response that includes rare neutralizing and highly protective antibodies that bind close to the threefold axes of the virus capsid, and larger numbers of non-neutralizing antibodies, many of which do not bind the viral capsid. The preference of germline genes and Vh/Vl pairs in the neutralizing antibody repertoire mirrors results from studies of donors after other viral infections^15–17^, suggesting that natural EV71 infection may expand similar repertoires of B cells. The majority of infection-induced neutralizing antibodies recognize a similar, conserved, epitope around the threefold axis but each interacts with the virion in the distinct configuration.

## Methods

### Ethics statement

The study protocol and informed consent were approved by the Research and Ethics Committee at the Chang Gung Medical Foundation. All subjects provided signed informed consent. The study was carried out in accordance with the Declaration of Helsinki and good clinical practice guidelines.

### Monoclonal antibodies

Fresh peripheral blood mononuclear cells were stained with fluorescent-labelled antibodies including Pacific blue anti-CD3 (clone UCHT1, catalogue number 558117, BD) (5 μg/ml), fluorescein isothiocyanate anti-CD19 (clone HIB19, catalogue number 555412, BD) (1:10 dilution in a 100 μl experimental sample), phycoerythrin-Cy7 anti-CD27 (clone M-T271, catalogue number 560609, BD) (1:20 dilution in a 100 μl experimental sample), allophycocyanin-H7 anti-CD20 (clone L27, catalogue number 641396, BD) (5 μg/ml) and phycoerythrin-Cy5 anti- CD38 (clone HIT2, catalogue number 555461, BD) (1:10 dilution in a 100 μl experimental sample). CD3^neg^CD20^neg^CD19^pos^CD27^hi^CD38^hi^ plasmablasts were gated (Supplementary Fig. 14a) and sorted as single cells. Heavy and light chain variable domains of single plasmablasts were cloned into their respective vectors and supernatants were collected from 293T cells co-transfected with the heavy chain and light chain vectors designed to express human IgG1^13^.

### Viruses, determination of viral titre and purification of virus

EV71 clinical strains including 98-4215, 99-1691, 00-2278, 07-72043, 11-96023, 12-96015 and 16-3443, prototype BrCr strain and Coxsackievirus A16 15-4126 were used in the study (Supplementary Table 4). Viruses were propagated in RD cells and viral titres determined by 50% tissue culture infectious dose (TCID_50_) assay using the Reed–Muench equation. The P1 gene of virus was sequenced for genotyping. Purified native EV71 was prepared by polyethylene glycol virus precipitation, ultracentrifugation pelleting, 10–35% potassium tartrate gradient purification and ultrafiltration^13^.

### Binding assay

Fixed and permeabilized virus-infected RD cells were blocked with saponin containing 3% BSA. Cells were incubated with antibody-containing cell culture supernatant with saponin, purified antibodies or serum in BD Perm/Wash™ buffer (antibody, 5 μg/ml; serum, 1:125 dilution) at 4 °C for 45 min. The controls included cells incubated with anti-influenza human IgG antibody 4A-14 (10 μg/ml)^15^ or PBS. After washing, cells were further incubated with fluorescein isothiocyanate-conjugated goat anti-human IgG secondary antibodies (2.5 μg/ml, Thermo Fisher Scientific) in BD Perm/Wash™ buffer at 4 °C for 30 min. After washing, cells were resuspended and analyzed with a BD FACSCanto flow cytometer (Supplementary Fig. 14b). At least 5000 events of EV71-infected cells were acquired and analyzed for each sample (Supplementary Fig. 14b).

### Enzyme-linked immunosorbent assays

Purified native virus preparations were absorbed to the wells of a microtiter plate (F96 Maxisorp NUNC Immuno plate). Nonspecific binding was blocked with PBS with 3% BSA. The antibody-containing cell culture supernatants or purified antibodies were applied and bound virus-specific antibodies were detected with horseradish peroxidase-conjugated rabbit anti-human IgG secondary antibody (0.25 μg/ml, Rockland Immunochemicals).

### Immunoprecipitation and western blot

For immunoprecipitation, Dynabeads^®^ Protein G (Thermo Fisher Scientific) was prepared following the protocol and incubated with the monoclonal antibody preparation. The beads-antibody complex was incubated with pre-cleared EV71-containing supernatants. The eluates were analyzed by western blot.

For western blot analysis, the eluates (antibody and antigen) were separated using SDS-PAGE and transferred to nitrocellulose membrane. After blocking, the membrane was probed with mouse anti-EV71 antibody (clone 422-8D-4C-4D, 1:1000 dilution, Sigma-Aldrich). Horseradish peroxidase-conjugated goat anti-mouse IgG antibody (1:10,000 dilution, Thermo Fisher Scientific) was used to detect bound antibodies.

### Neutralization assay

Neutralizing activities of sera against EV71 and other enteroviruses were evaluated by the protocol described previously^42^. Briefly, serially diluted samples were mixed with an equal volume of 100 TCID_50_ virus preparation and incubated at 37 °C for 2 h. The mixture was incubated with seeded RD cells at 37 °C for 4 days. Cytopathic effect was checked before and after crystal violet staining of attached cells on the plates. For each experiment, the cell control, positive serum/antibody control and virus back-titration were setup. All samples were assayed in triplicate. When the cytopathic effect is observed in 1 TCID_50_ in the control wells of virus back-titration, the neutralization titre is determined as the reciprocal of the sample dilutions that completely prevented the cytopathic effect in all three triplicate wells.

The neutralization assay was performed to examine the activities of human IgG monoclonal antibodies. Briefly, serially diluted antibodies in virus dilution medium (DMEM/10% FBS/Penicillin and Streptomycin) were incubated with 100 TCID_50_ of virus preparation at 37 °C for 2 h. The antibody-virus mixture was added into confluent monolayers of RD cells seeded the day before and incubated at 37 °C for 4 days. At the end of incubation, the cells were examined for the development of cytopathic effect and the viability of cells determined by the MTT (3-(4,5-dimethylthiazol-2-yl)-2,5-diphenyl tetrazolium bromide) assay. The optical density of the supernatant was measured at OD570 on a microplate reader. At each dilution of a given sample, percent infection was calculated as [1 − (value for antibody − value for virus only)/(value for cell only − value for virus only))] × 100. The concentration of antibody that inhibited infection by 50% (EC50) was calculated by nonlinear regression analysis, performed in GraphPad Prism software.

### Selection of EV71 mutants with monoclonal antibodies

Escape mutants of EV71 were selected in a protocol similar to that described previously^13^. Briefly, a preparation of monoclonal antibody at 25 μg/ml was incubated with an optimal amount of plaque-purified EV71 (50 TCID_50_ times neutralization titre against tested monoclonal antibody) and the mixture was then added to a confluent monolayer of RD cells in viral growth medium (DMEM/2% FBS/Penicillin and Streptomycin) at 37 °C for 4 days. After first cycle of incubation, the cells and supernatant were collected, freeze-thawed, and then re-infected RD cells. Once cytopathic effect of cells was observed at first or second re-infection cycles, the virus solution was harvested and the virus was plaque-purified. Plaque-purified antibody-resistant mutants were confirmed by loss of binding and neutralization with antibody.

Escape mutants from 11-96023 (genotype C4), 00-2278 (genotype B4) viruses were selected in the presence of neutralizing antibody in vitro. The mutants showed infectivity for RD cell line similar to that of parent strain (~7.9 × 10^8^ TCID50/ml for 11-96023 and ~7.4 × 10^8^ for 00-2278 mutants).

### Site-directed mutagenesis of the infectious clone

Site-directed mutagenesis was carried out on an infectious cDNA clone of genotype B5 EV71 (strain TW-1745, GenBank accession number KT354870.1) (kindly provided by Jen-Ren Wang, National Cheng Kung University, Taiwan)^13^. The site-specific mutation in the infectious clone was generated using the QuikChange site-directed mutagenesis Kit (Agilent). Plasmid DNAs of mutant clones were linearized by MluI (New England Biolabs), phenol/chloroform extraction, ethanol precipitation and then dissolved in RNase-free water. The linear DNA template was used for in vitro transcription to generated viral RNAs (Thermo Fisher Scientific). After precipitation and purification, the viral RNAs were transfected into RD cells by using Lipofectamine^®^ 2000 reagent (Thermo Fisher Scientific) and the viral supernatant was harvested 3–4 days later. After confirming the site-specific mutation in the mutated virus, the viral titre was determined by TCID_50_ assay.

### Pre- and post-attachment assay

For pre-attachment neutralization assay, serially diluted antibodies were incubated with 100 TCID_50_ of virus preparation at 37 °C for 1 h. Pre-chilled antibody-virus mixture was added to a confluent monolayer of RD cells plated the day before and incubated at 4 °C for 1 h. After washing with cold virus dilution medium to remove unbound virus, cells were incubated at 37 °C for a further 5 days. For post-attachment neutralization assay, pre-chilled 100 TCID_50_ of virus preparation was incubated with a confluent monolayer of RD cells plated the day before at 4 °C for 1 h. After washing with cold virus dilution medium to remove unbound virus, cells were immediately incubated in serially diluted antibodies at 37 °C for 1 h. The cells were washed with cold virus dilution medium and incubated at 37 °C for further 5 days. At the end of incubation, the viability of cells was determined by the MTT assay.

### Animal studies

Animal experiments were performed in accordance with the protocol approved by the Institutional Animal Care and Use Committee in the Chang Gung University, Taiwan. Experiments were carried out in accordance with the ‘Guide for the care and use of laboratory animals’, the recommendations of the Institute for Laboratory Animal Research and Association for Assessment and Accreditation of Laboratory Animal Care International standards. hSCARB2-transgenic C57BL/6 mice were housed at room temperature 20–23 °C with a relative humidity between 55 and 60% and kept under a 12-h light:12-h dark cycle. Three-week-old specific pathogen-free hSCARB2-transgenic C57BL/6 mice were randomly distributed in experimental groups of animals. Both males and females were used for these studies. They were anesthetized under aseptic conditions prior to virus infection. In the prophylaxis experiment, each group received a dose of intraperitoneal antibodies or PBS administration 24 h before intraperitoneal infection with EV71 (10 LD50). In the therapeutic experiment, each group received a dose of intraperitoneal antibodies or PBS administration 12, 24, 36, 48 or 72 h after intraperitoneal infection with EV71 (10 LD50). Mice were weighed, their motor functions were scored and mice with 20% weight loss were humanely killed.

### Preparation of Fab 38-3-11A

Fab 38-3-11A for cryo-EM experiments was generated from IgG 38-3-11A by papain digestion following the protocol of Pierce Fab Preparation Kit (Thermo Fisher Scientific).

### Expression and purification of Fab 38-1-10A

Gene blocks encoding the heavy and light chain variable domains of Fab 38-1-10A were ordered from Integrated DNA Technologies and cloned directly into the expression vectors using Ligation Independent Cloning following the protocol of In-Fusion Cloning Kit (Bimake), with the heavy chain gene cloned into the vector pOPINVH (with the sequence encoding a C-terminal 6*His tag) and the light chain gene into pOPINVL^43^. HEK293T cells (ATCC CRL-11268) were transfected with both the heavy and light chain recombinant plasmids. The culture supernatant was harvested and dialysed in 23 mM Na_2_HPO_4_, 1.7 mM NaH_2_PO_4_, pH 8.0, 250 mM NaCl at 4 °C. The Fab was then purified with a 5 mL nickel columns (GE) and further purified using a Superdex 75 HiLoad 16/60 column (GE), eluted in 20 mM Tris, 200 mM NaCl, pH 7.4. The purified protein was concentrated using a 10 KD centrifugal filter (Sigma-Aldrich).

### Crystallization of Fab 38-1-10A

The concentration of Fab 38-1-10A used for crystallization was 45 mg/ml. Crystals were grown in CrystalQuick X plates (Greiner Bio-One) at 20 °C using the sitting-drop vapour-diffusion method with 100 nl Fab plus 100 nl precipitant dispensed with a Cartesian robot^44,45^. Images of drops were taken using a Formulatrix imager. The best crystals were grown in the condition containing 20% (v/v) PEG 6000, 0.1 M citrate, pH 4.0, 1 M lithium chloride.

### X-ray diffraction data collection and structure determination

A number of crystals were immersed in the reservoir solution supplemented with either 25% (v/v) glycerol or ethylene glycol and frozen in liquid nitrogen. X-ray diffraction data were collected at I03 beamline at Diamond Light Source (Didcot, Oxford) using a Pilatus 6M detector (0.1° rotation, beam size 80 × 20 μm, exposure time 0.02 s per frame and 100% transmission). Data were indexed and integrated with Xia2-3dii^46^ and data from nine crystals with the same space group and similar unit cell were merged together. Molrep^47^ was used to perform molecular replacement. REFMAC^48^ and Phenix^49^ were used to refine the structure. Model building was performed in COOT^50^. Data collection and refinement statistics are shown in Supplementary Table 2.

### Virus production and purification for cryo-EM

EV71 genotype B2 (strain MS742387) was used to infect human RD cells. Three days after infection, virus was harvested and 8% (w/v) PEG 6000 and 0.5% (v/v) NP40 added to precipitate virus. The sample was centrifuged at 3500 × *g* for 1 h at 4 °C and the pellet stored at −80 °C until needed. The thawed pellet was suspended in buffer (50 mM HEPES, pH 7.4, 200 mM NaCl, 0.5% (v/v) NP40) and centrifuged at 3500 × *g* for 30 min at 4 °C to remove cell debris. Virus particles in the supernatant were pelleted through a 2 ml 30% (w/v) sucrose cushion (in 50 mM HEPES, pH 7.4, 200 mM NaCl) at 105,000 × *g* for 3 h at 4 °C using a SW32 rotor (Beckman). The pellet was suspended in buffer (50 mM HEPES, pH 7.4, 200 mM NaCl, 0.5% (v/v) NP40) and centrifuged at 12,000 × *g* for 30 min at 4 °C twice to remove insoluble material. The supernatant was then laid on the top of a 15–45% sucrose gradient (in 50 mM HEPES, pH 7.4, 200 mM NaCl) and centrifuged at 105,000 × *g* for 3 h at 4 °C using a SW32 rotor (Beckman). EV71 full particles were harvested and purified with a Zeba desalting column (Thermo Fisher Scientific) to remove sucrose, then concentrated using 100 KDa centrifugal filters (Sigma-Aldrich). The concentration of virus particles was measured using a NanoDrop spectrophotometer.

### Cryo-EM grid preparation

EV71 virions (~0.8 mg/ml) were incubated with Fab 38-1-10A (5 mg/ml) and 38-3-11A (9.5 mg/ml) separately on ice for 5–20 min, with a molar ratios of Fab:EV71 capsid of ~600 and 1200 to 1, respectively. Four microlitres of the sample was applied to a glow-discharged ultrathin carbon Lacey grid (Agar Scientific), blotted by filter paper from the back of the grid (blotting time ~3 s) and vitrified by plunging into liquid ethane or ethane/propane mix (50%:50%) using a manual plunger (Max Planck Institute of Biochemistry, Germany).

### Cryo-EM data collection

Cryo-EM data for the EV71/Fab 38-1-10A complex were collected using a 300-kV Tecnai Polara microscope (FEI) with a K2 Summit detector mounted after a GIF Quantum energy filter (Gatan), and data for EV71/Fab 38-3-11A complex were collected using a 300-kV Titan Krios microscope and Falcon III detector (Thermo Fisher Scientific). For the EV71/Fab 38-1-10A complex, data were recorded as movies (40 frames, each 0.2 s) in super-resolution mode using SerialEM^51^ with a defocus range of −0.5 to −2.5 μm. The calibrated magnification was ×37,037, corresponding to a pixel size of 1.35 Å. The dose rate was ~4 e^−^/Å^2^/s, resulting in a total electron dose of 30 e^−^/Å^2^. For the EV71/Fab 38-3-11A complex, movies (30 frames, each 0.25 s) were recorded in linear mode using EPU (Thermo Fisher Scientific) (defocus range: −1 to −3.4 μm, calibrated magnification: ×130,000, calibrated pixel size: 1.05 Å, dose rate: ~5 e^−^/Å^2^/s and total dose: 41 e^−^/Å^2^).

### Cryo-EM data processing

Frames of each movie were aligned and combined using MotionCorr2^52^ and contrast transfer function (CTF) parameters determined with CTFFIND4^53^. Micrographs with significant drift or astigmatism were discarded. Particles were automatically picked using ETHAN^54^ or Warp^55^. The structure was calculated with Relion 3.0 following the gold-standard refinement procedure^56^. Reference-free 2D-class averaging was performed with particles being classified into 20 classes. Based on the 2D classification results, good particles were selected and used for reference-based 3D classification with particles being classified into four classes. The initial reference model was generated by filtering the crystal structure of EV71 (PDB: 3VBS^7^) to 50 Å resolution. The best map calculated from 3D classification was used as the initial reference model for 3D refinement after which all complexes were further processed using the post-processing function in Relion to sharpen the maps, by applying a negative B-factor to each map. Post-processing and CTF refinement were performed in Relion 3.0 to further improve the density maps. The final density map of EV71_Fab-38-1-10A was calculated using 10074 particles from 324 micrographs, with an overall resolution of 2.7 Å, and the map of EV71_Fab-38-3-11A was calculated using 14430 particles from 2084 micrographs, with an overall resolution of 2.8 Å. These resolutions were based on the “gold” standard Fourier shell correlation (threshold = 0.143 criterion) between two independent “half maps”^57^.

### Model building

The crystal structures of EV71 (PDB: 3VBS^7^) and Fab 38-1-10A were fitted into the density maps of these two complexes in COOT^50^. The amino acid sequence of Fab 38-1-10A was changed to get the correct sequence of Fab 38-3-11A. The model was further improved using Phenix.real_space_refine^58^. Refinement statistics are given in Supplementary Table 3. The residues forming the EV71-Fab interfaces were identified with PISA^59^. Roadmaps were calculated using Rivem^60^. Other structural figures were prepared with PYMOL^61^ and CHIMERA^62^.

### Statistics

The TCID_50_ in the RD cell line for each virus were calculated by Reed–Muench method with SPSS. The EC50s for each neutralizing antibody were calculated by nonlinear regression analysis (log concentration vs. response, variable slope) in GraphPad Prism. A *p* value of less than 0.05 was considered significant. Graphs were presented by Microsoft Excel and GraphPad Prism software.

### Reporting summary

Further information on research design is available in the Nature Research Reporting Summary linked to this article.

## Supplementary information

Supplementary Information

Peer Review File

Reporting Summary

## Data Availability

All the data supporting the findings of this study are available within the paper and Supplementary Information. Requests for antibody material should be addressed to K.-Y.A.H. The coordinates for the crystal structure of Fab 38-1-10A (along with the structure factors), the EV71/Fab 38-1-10A complex and the EV71/Fab 38-3-11A complex are deposited with PDB codes 6Z3K, 6Z3Q and 6Z3P, respectively. The EM reconstructions for the Fab 38-1-10A and Fab 38-3-11A complexes are deposited with EMDB, codes EM-11062 and EM-11061. Source data are provided with this paper.

## Source data

Source Data
