# Supplementary material for: Structural and functional analysis of protective antibodies targeting the threefold plateau of enterovirus 71
Source: Nat Commun. 2020 Oct 16;11:5253. doi: 10.1038/s41467-020-19013-3 (PMC7567869; doi:10.1038/s41467-020-19013-3)
Supplement: Supplementary file 1 — Supplementary Information [file 41467_2020_19013_MOESM1_ESM.pdf]

Supplementary information for

Structural and functional analysis of protective antibodies targeting the 3-fold plateau of Enterovirus 71

Huang et al.

Supplementary Figures 1-14

Supplementary Tables 1-4

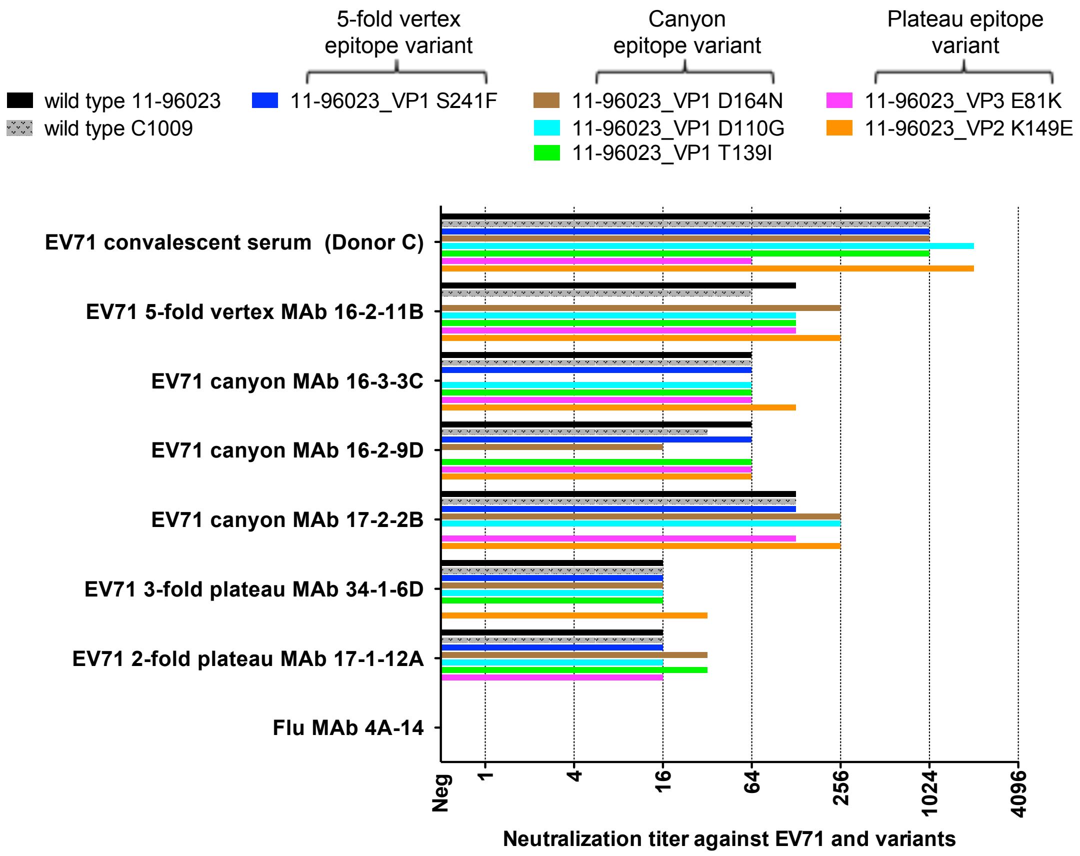

Supplementary Figure 1 | Neutralizing titer of donor C's convalescent serum (day 9 after illness onset) against wild type EV71 and escape variants. EV71 C1009 strain was isolated from donor C and the convalescent serum showed a strong neutralization titer (1:1024) to C1009. The serum was tested with another wild type EV71 11-96023 and its epitope variants[1] (selected *in vitro* with neutralizing monoclonal antibodies). Epitope variants of EV71 11-96023 that contain single amino acid mutation[1] in the 5-fold vertex epitope (VP1 S241F), canyon epitope (VP1 D164N, VP1 D110G, or VP1 T139I), 3-fold plateau epitope (VP3 E81K) and 2-fold plateau epitope (VP2 K149E) were included. Control neutralizing antibodies 16-2-11B, 16-3-3C, 16-2-9D, 17-2-2B, 34-1-6D and 17-1-12A (starting at 40 μg/ml) lost reactivity to the corresponding epitope variant. Influenza neutralizing antibody 4A-14[2] was included as the control in the experiment.

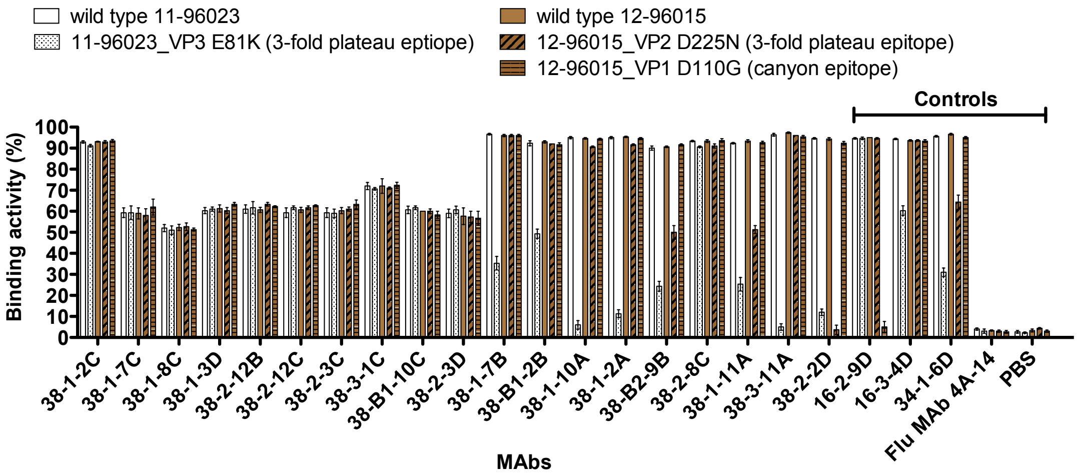

Supplementary Figure 2 | Binding activities of EV71-reactive monoclonal antibodies derived from donor C to wild type and EV71 variants, measured by flow cytometry. Data are presented as the mean± standard error of the mean and represent three independent experiments (n=3). Error bars represent the standard error of mean. EV71-neutralizing human antibodies 16-2-9D (capsid canyon-binding), 16-3-4D and 34-1-6D (3-fold plateau-binding)[1] and influenza H7-neutralizing human antibody 4A-14[2] were included as antibody controls.

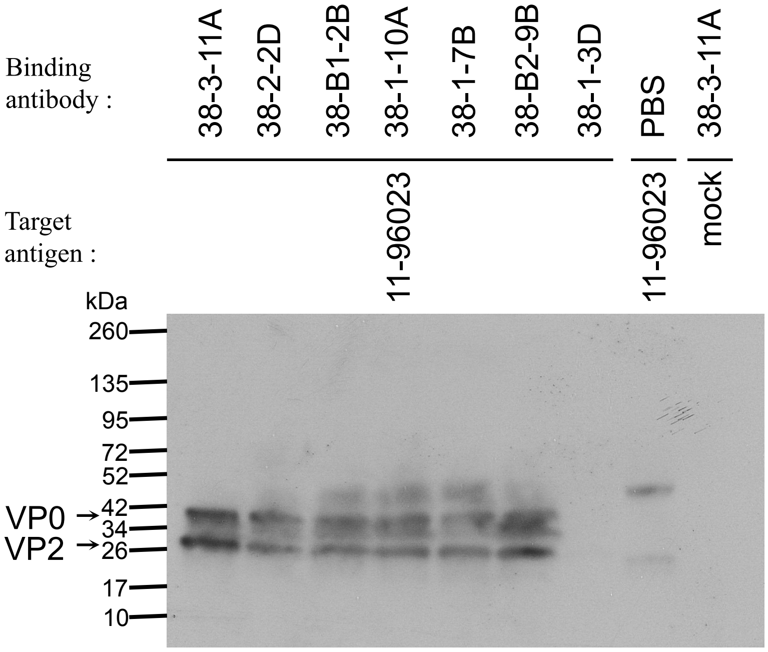

Supplementary Figure 3 | Immunoprecipitation of EV71 virion by monoclonal antibodies derived from donor C. Immunoprecipitated EV71 capsids were detected by immunoblot analysis using anti-VP0/VP2 monoclonal antibody MAB979.

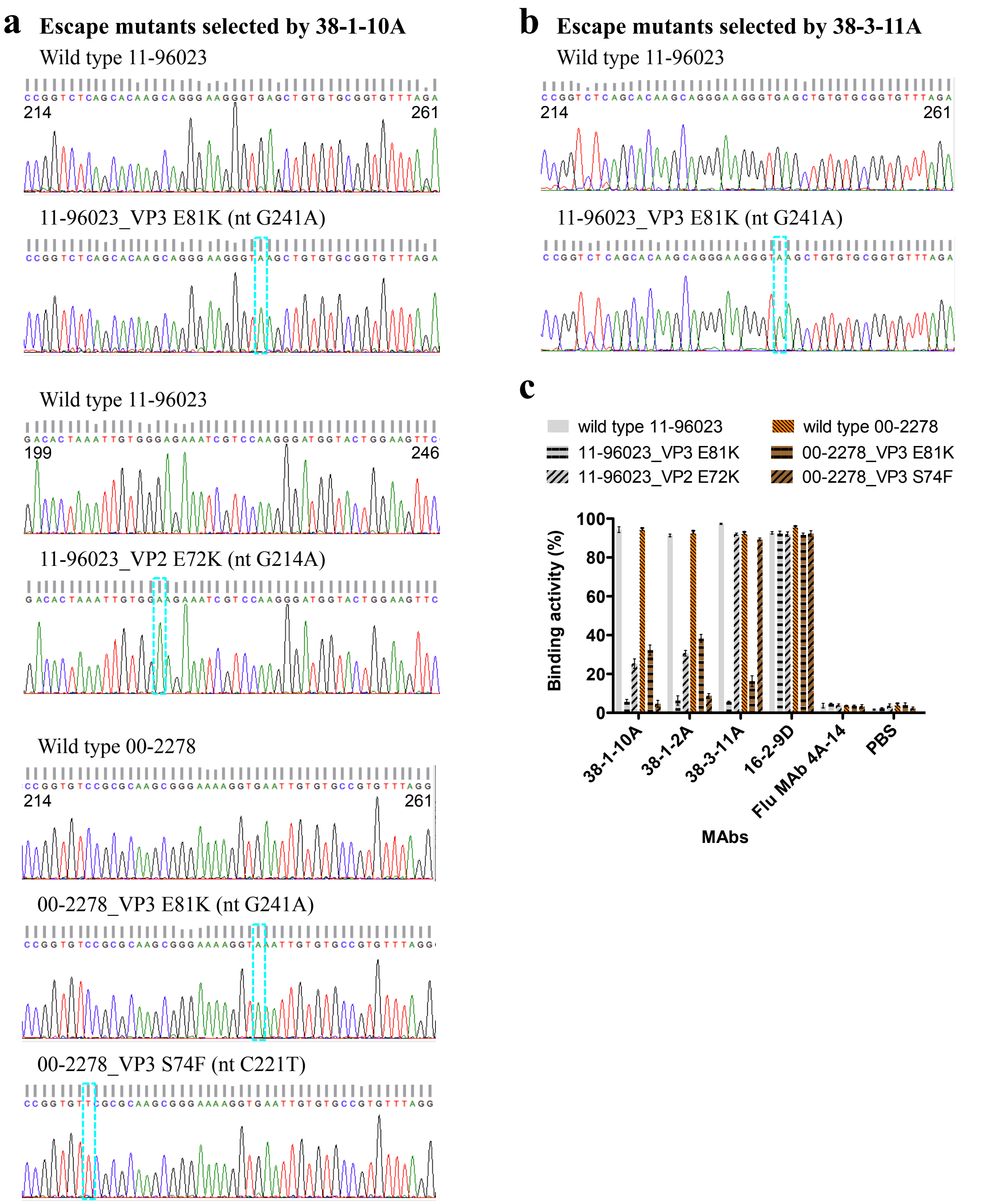

Supplementary Figure 4 | Escape mutants of EV71 selected with neutralizing antibodies. a, EV71 11-96023 (genotype C4) escape mutants selected with 38-1-10A had a single VP3 E81K or VP2 E72K substitution. EV71 00-2278 (genotype B4) escape mutants selected with 38-1-10A had a single VP3 E81K or VP3 S74F substitution. **b**, EV71 11-96023 (genotype C4) escape mutants selected with 38-3-11A had a single VP3 E81K substitution. **c**, Binding activities of neutralizing antibodies to

wild type and EV71 escape mutants, measured by flow cytometry. Data are presented as the mean± standard error of the mean and represent three independent experiments (n=3). Error bars represent the standard error of mean. EV71-neutralizing human antibodies 16-2-9D (capsid canyon-binding)[1] and influenza H7-neutralizing human antibody 4A-14[2] were included as antibody controls.

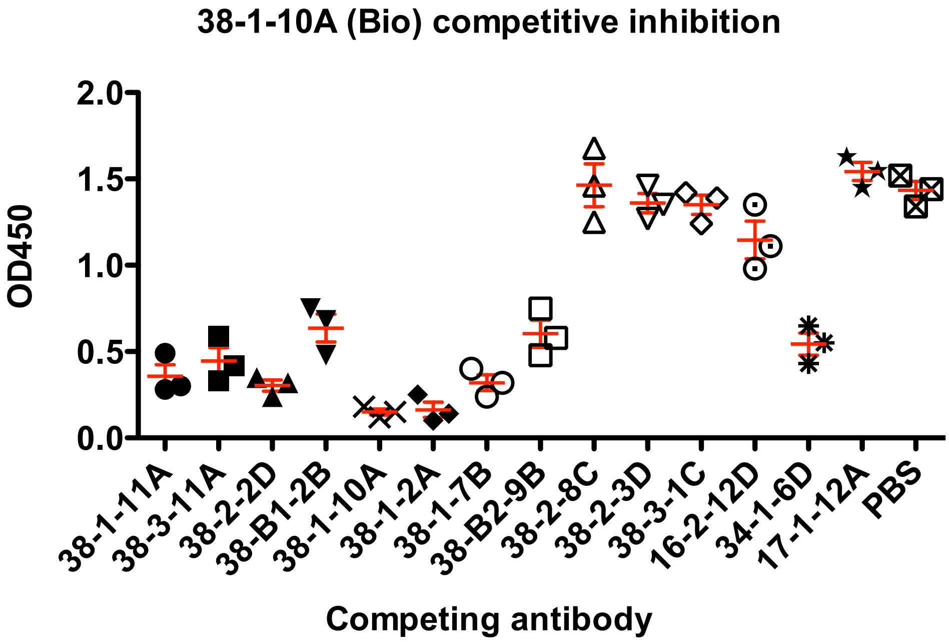

Supplementary Figure 5 | Competitive binding of EV71-specific antibodies to EV71 11-96023 antigen. A 10-fold molar excess of unlabeled antibody competed with a biotinylated antibody 38-1-10A for the binding to purified 11-96023 viruses in the ELISA assay. Control antibodies, 16-2-12D (canyon-binding), 34-1-6D (3-fold plateau-binding) and 17-1-12A (2-fold plateau-binding), were included in the assay[1]. Data are presented as means plus 95% confidence intervals of three independent experiments (n=3). Error bars represent the 95% confidence interval.

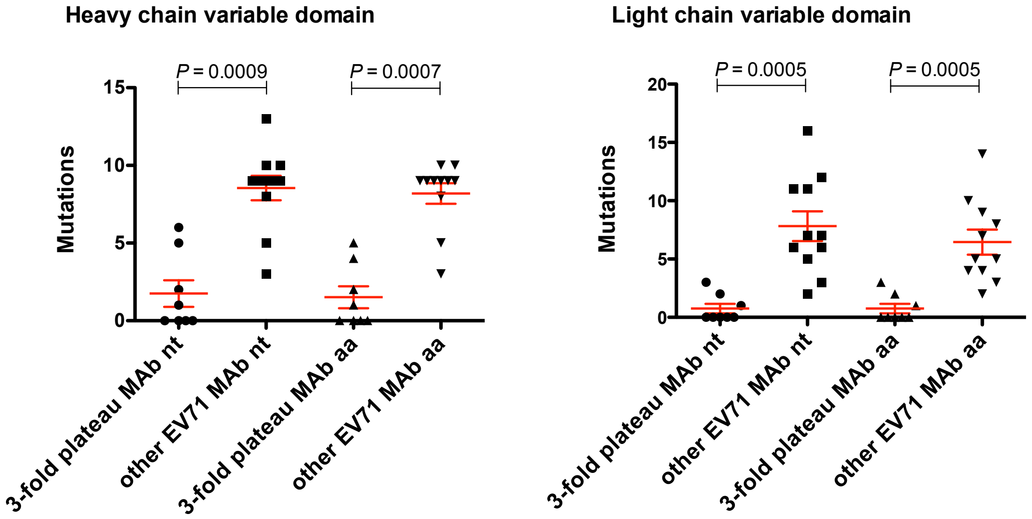

Supplementary Figure 6 | Somatic mutations of 3-fold plateau-targeting and other EV71 capsid-reactive antibodies. Among EV71 capsid-reactive antibodies, significantly fewer mutations are found in the heavy and light chain variable domain of the 3-fold plateau-binding group (antibodies including 38-1-11A, 38-3-11A, 38-2-2D, 38-B1-2B, 38-1-10A, 38-1-2A, 38-1-7B and 38-B2-9B, n=8) than those of other EV71 antibodies (antibodies including 38-2-8C, 38-2-3D, 38-1-2C, 38-B1-10C, 38-1-3D, 38-1-7C, 38-3-1C, 38-2-3C, 38-1-8C, 38-2-12B and 38-2-12C, n=11). The lines represent mean ± standard error of the mean. Error bars represent the standard error of mean. The difference in the numbers of mutations between two groups was examined using a two-tailed Mann–Whitney test. nt, non-silent nucleotide mutation; aa, amino acid mutation.

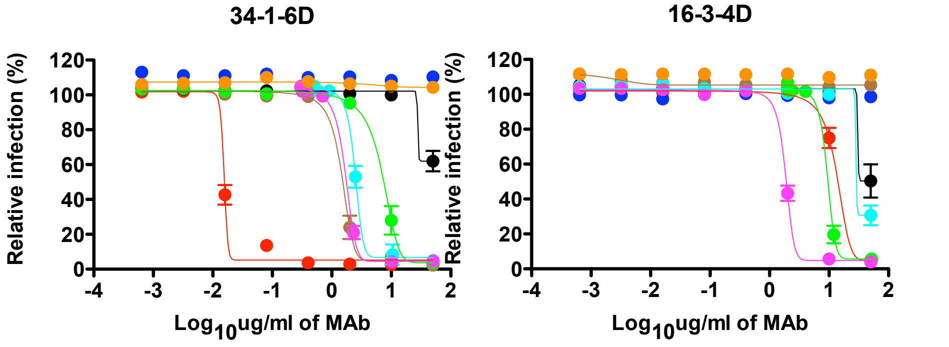

	34-1-6D	16-3-4D
BrCr (Genotype A)	EC50=0.012 µg/ml, R=0.9879	EC50=16.92 µg/ml, R=0.9943
98-4215 (year 1998 C1)	Negative	Negative
99-1691 (year 1999 C2)	Negative	Negative
16-3443 (year 2016 C4)	EC50=1.292 µg/ml, R=0.9762	Negative
07-72043 (year 2007 C5)	EC50=2.370 µg/ml, R=0.9845	EC50= non-available (95% CI very wide)
00-2278 (year 2000 B4)	EC50=8.464 µg/ml, R=0.9779	EC50=7.504 µg/ml, R=0.9646
12-96015 (year 2012 B5)	EC50=1.187 µg/ml, R=0.9747	EC50=1.935 µg/ml, R=0.9854
15-4126 (Cox A16)	Negative	Negative

Supplementary Figure 7 | Neutralizing activities of antibodies 34-1-6D and 16-3-4D against a panel of EV71 clinical strains in 1998-2016 and the prototype BrCr strain. Data are presented as mean ± standard error of the mean and represent measurements from three independent experiments (n=3). Error bars represent the standard error of mean. The concentration of antibody that inhibited infection by 50% (EC50) was determined using nonlinear regression (log concentration vs. response, variable slope) in GraphPad Prism. Coxsackievirus A16 was included in the neutralization assay as a control.

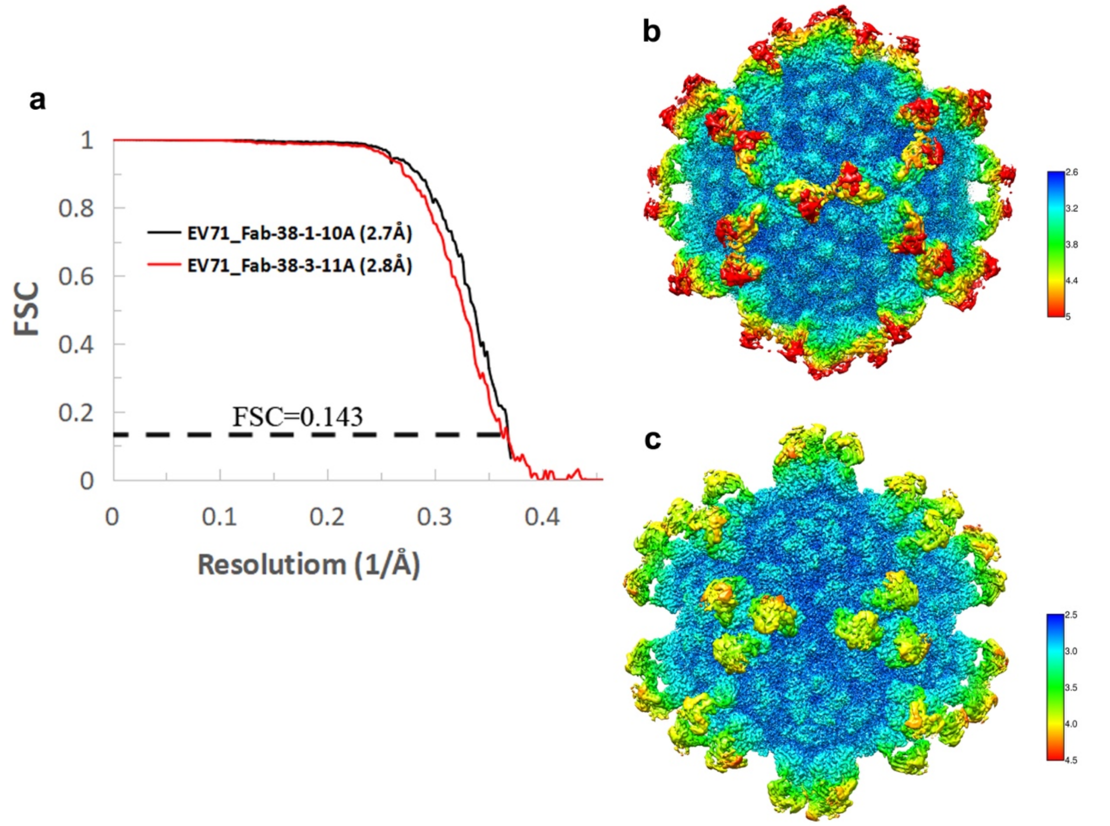

Supplementary Figure 8 | Overall quality of the EM maps. a, The gold standard Fourier shell correlation curves of the final maps showing resolutions (for FSC cut-off of 0.143) of 2.7 Å for the EV71/38-1-10A complex (black line) and 2.8 Å for the EV71/38-3-11A complex. **b**, **c**, EM maps are coloured by local resolution as calculated by Xmipp MonoRes for: EV71/38-1-10A complex (**b**) and EV71/38-3-11A complex (**c**).

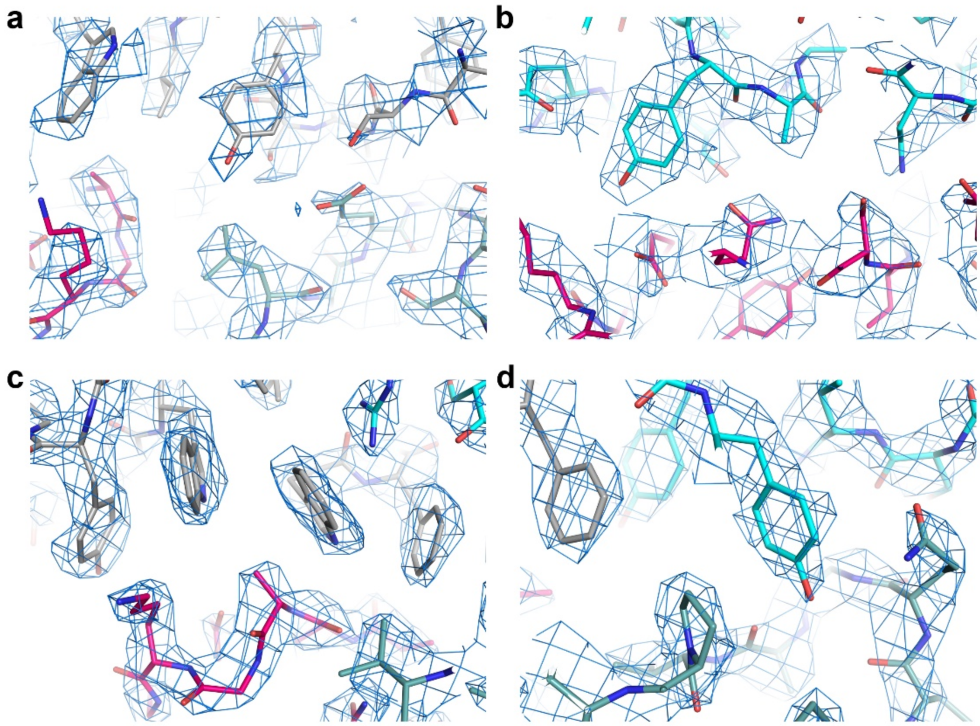

Supplementary Figure 9 | Electron density maps at the viral-Fab interface. a, Electron density at the EV71/Vh and (**b**) EV71/Vl interface of the EV71/38-1-10A complex. **c**, **d**, Electron density at the interface of EV71/Vh and EV71/Vl of the EV71/38-3-11A complex, respectively. The contour level for 38-1-10A in (**a**) and (**b**) is 40% of the contour level for the virus. Residues of VP3 are shown as red sticks and those from VP2 of a neighbouring protomer as teal sticks. Residues of Vh and Vl domains of the Fabs are drawn as grey and cyan sticks, respectively.

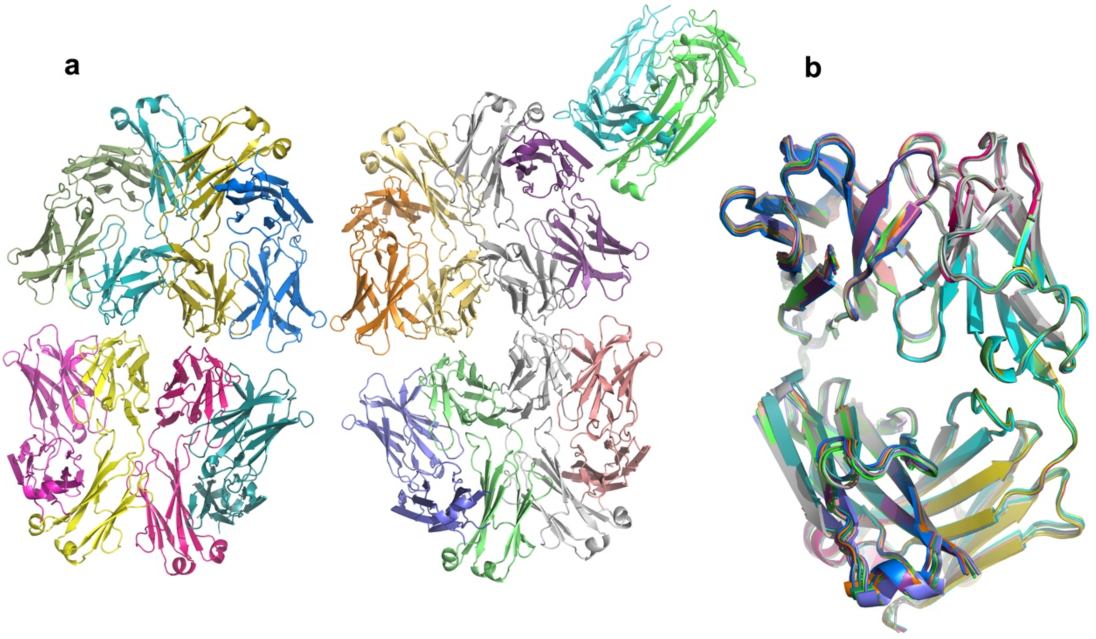

Supplementary Figure 10 | Crystal structure of 38-1-10A. a, The nine 38-1-10A Fab molecules in the crystallographic asymmetric unit. **b**, Superimposition of the nine Fab molecules.

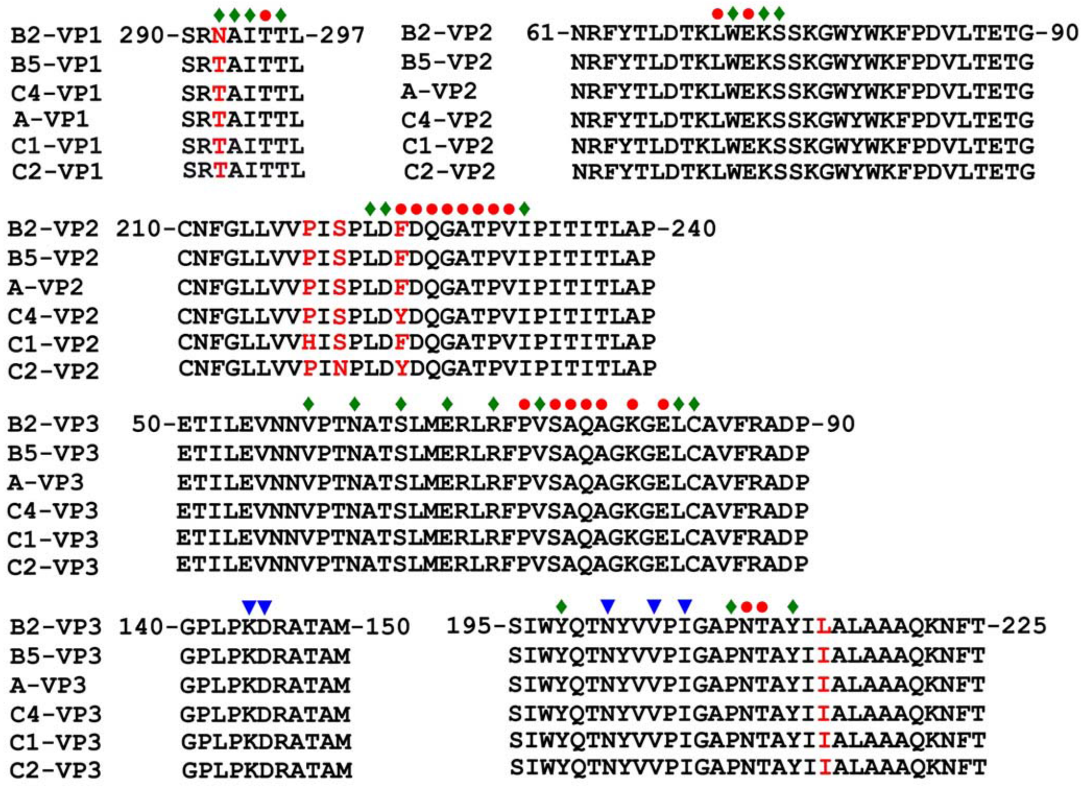

Supplementary Figure 11 | Comparison of epitope residues recognized by 38-1-10A and 38-3-11A antibodies. Amino acids sequences of four EV71 genotypes (B2, AAB39969; B5, AFN20475; A, AFN20470; C1, AFN20476; C2, AAD22046; C4, AFN20480) are aligned. Solvent accessible surface of the capsid residues sheltered by 38-1-10A are marked with green diamonds, those sheltered by 38-3-11A with blue triangles, and residues sheltered by both with red disks. Non-conserved residues are shown in red.

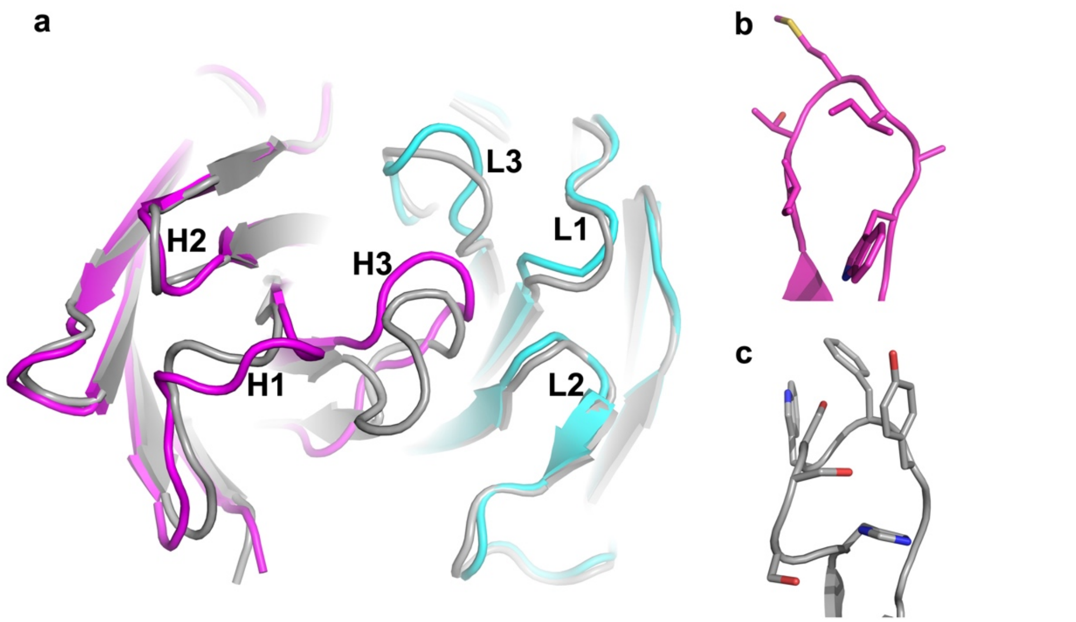

Supplementary Figure 12 | Overlay of the VhVl domains of 38-3-11A and 38-1-10A. a, The heavy chain and light chains of 38-1-10A are coloured in magenta and cyan respectively, 38-3-11a is shown in grey. **b**, **c**, CDR3 structures of 38-1-10A (**b**) and 38-3-11A (**c**).

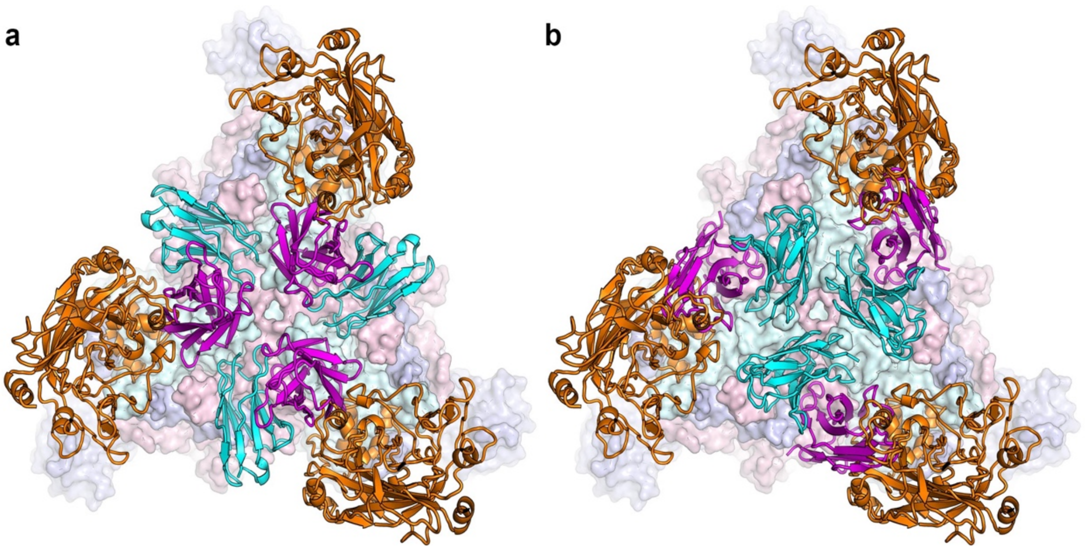

Supplementary Figure 13 | Comparing the binding of Fab with receptor. a, Overlap of EV71/38-1-10A with EV71/SCARB2. **b**, Overlap of EV71/38-3-11A with EV71/SCARB2. Three viral protomers and the bound Fab and receptor around a 3-fold axis are shown. The virus is drawn and coloured as in Fig. 1a, the Fab heavy chains are in magenta, the light chains in cyan and the receptor as orange ribbons.

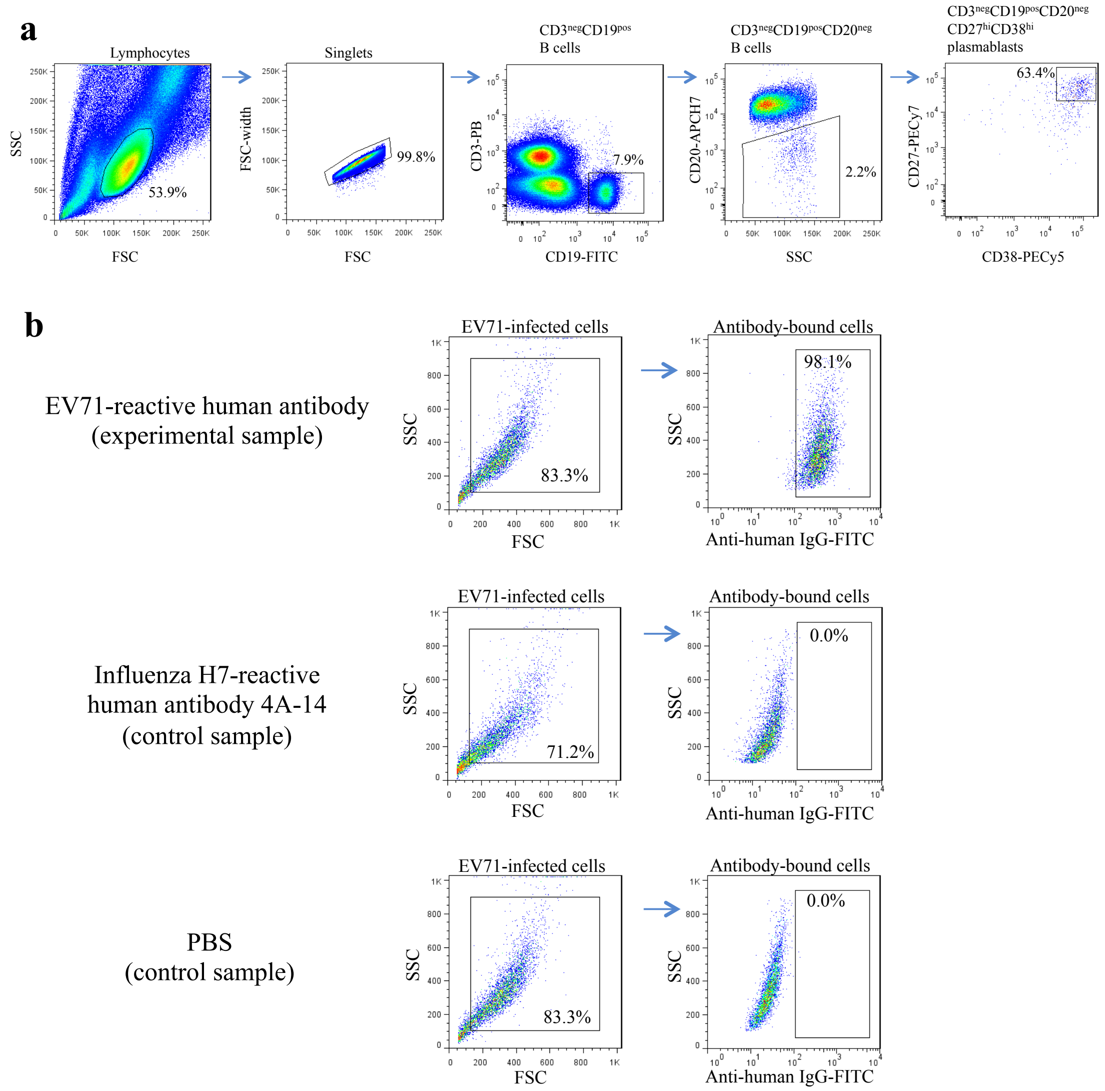

Supplementary Figure 14 | Gating strategy for plasmablasts sorting and EV71-specific antibody binding assay. a, Gating strategy of plasmablasts. Fresh peripheral blood mononuclear cells were stained with fluorescent-labeled antibodies PB anti-CD3, FITC anti-CD19, APCH7 anti-CD20, PECy7 anti-CD27, and PECy5 anti-CD38. $CD3^{neg}CD20^{neg}CD19^{pos}CD27^{hi}CD38^{hi}$ plasmablasts were gated and sorted as single cells for production of human monoclonal antibodies. **b**, Gating strategy of antibody-bound EV71-infected cells in the binding assay. This flow cytometry-based binding assay was used to characterize plasmablast-derived EV71-specific monoclonal as shown in Fig. 1b. In the binding assay, EV71-infected RD cells were

incubated with EV71-specific monoclonal antibody-containing cell culture supernatant or purified EV71-specific antibodies. The controls included cells incubated with anti-influenza human IgG antibody 4A-14 or PBS. Antibody-bound cells were detected with FITC-conjugated goat anti-human IgG secondary antibodies. In the binding assay, infected RD cells were included using FSC/SSC gate and $FITC^{pos}$ (antibody-bound) cells were gated and analyzed.

Supplementary Table 1 | Details of 19 EV71 capsid-reactive monoclonal antibodies variable domain sequences isolated from donor C

MAb	Vh	Jh	Dh	rf	V_H junction sequence	V_H Mut (non-silent)	aa Mut	λ/κ	Vl	Jl	V_L Junction Sequence	V_L Muts (non-silent)	aa Mut
38-1-2C	3-21*01	5*02	3-10*01	2	CARGYYGSGRYKRYNWFDPW	7 (3)	3	κ	1-39*01 or 1D-39*01	5*01	CQQSYSTPLTF	2 (2)	2
38-1-7C	3-30*03 or 18 or 3-30-5*01	4*02	5-18*01	3	CTKDRVAYSYGPTRVDYW	9 (8)	8	κ	1-39*01 or 1D-39*01	1*01	CQQTYSIPRTF	9 (7)	7
38-1-8C	3-30*03 or 18 or 3-30-5*01	4*02	5-18*01	3	CTKDRVAYSYGPTRVDYW	11 (9)	9	κ	3-20*01	2*01	CHHYDSSSMYTF	6 (5)	5
38-1-3D	3-30*03 or 18 or 3-30-5*01	4*02	5-18*01	3	CTKDRVAYSYGPTRVDYW	12 (10)	9	κ	3-15*01	4*01	CQQYNNWPLTF	13 (11)	8
38-2-12B	3-30*03 or 18 or 3-30-5*01	4*02	5-18*01	3	CTKDRVAYSYGPTRVDYW	11 (9)	9	κ	4-1*01	3*01	CQQYYGAPLTF	17 (12)	10
38-2-12C	3-30*03 or 18 or 3-30-5*01	4*02	5-18*01	3	CTKDRVAYSYGPTRVDYW	11 (9)	9	κ	1-39*01 or 1D-39*01	3*01	CQQSYSTPLTF	11 (6)	4
38-2-3C	3-30*03 or 18 or 3-30-5*01	4*02	5-18*01	3	CTKDRVAYSYGPTRVDYW	12 (10)	10	λ	3-25*03	2*01 or 3*01	CQSADSSNLYAVF	15 (11)	9
38-3-1C	3-30*03 or 18 or 3-30-5*01	4*02	5-18*01	3	CTKDRVAYSYGPTRVDYW	11 (9)	9	κ	1-17*03	4*01	CLQHYSYPLTF	9 (6)	4
38-B1-10C	3-30*03 or 18 or 3-30-5*01	4*02	5-18*01	3	CTKDRVAYSYGPTRVDYW	11 (9)	9	κ	3-20*01	1*01	CHQYARSPWTF	19 (16)	14
38-2-3D	3-74*01 or 03	6*02	1-14*01	3	CARDQNYGVDVW	10 (5)	5	κ	1-39*01 or 1D-39*01	1*01	CQQSFSTPQTF	3 (3)	3
38-1-7B	4-38-2*02	6*02	5-18*01	1	CGRDTDYYGMDVW	1 (0)	0	κ	3-20*01	1*01 or 2*02	CQQYGSSPRTF	2 (0)	0
38-B1-2B	4-38-2*02	5*02	3-22*01	2	CARVGSSGYLGWFDPW	2 (2)	2	κ	1-39*01 or 1D-39*01	2*01	CQQSYSTPYTF	3 (3)	3
38-1-10A	4-39*01	5*02	3-22*01	3	CAREITMIAWFDPW	0 (0)	0	κ	1-6*01	4*01	CLQDYNYLLTF	1 (1)	1
38-1-2A	4-39*01	5*02	3-22*01	3	CAREITMIAWFDPW	0 (0)	0	κ	1-6*01	4*01	CLQDYNYLLTF	0 (0)	0
38-B2-9B	4-39*07	6*02	1-14*01	3	CAGQTRYYGMDVW	8 (6)	5	κ	3-20*01	5*01	CQQYGSSEITF	3 (2)	2
38-2-8C	4-59*01	6*02	3-9*01	2	CARAPRPYSDILTGHYHYYYYYGMDVW	18 (13)	10	λ	1-44*01	1*01	CAAWDDSLNDYVF	11 (7)	5
38-1-11A	5-51*01	6*01	3-16*02	3	CARRGGSGILHGMDVW	6 (5)	4	κ	1-39*01 or 1D-39*01	2*01	CQQSYSTPMYTF	0 (0)	0
38-3-11A	5-51*01 F	6*02 F	6-13*01 F	1	CARLHSSSWFYGMDVW	0 (0)	0	κ	1-39*01 or 1D-39*01	3*01	CQQSYSTPRTF	0 (0)	0
38-2-2D	7-4-1*02	6*02	2-21*01	3	CARALSAYYYGMDVW	4 (1)	1	κ	1-39*01 or 1D-39*01	5*01	CQQSYSTPRTF	0 (0)	0

* The number of nucleotide mutations in the heavy and light chain variable domains and the number of non-silent mutations (shown in parentheses). The variable domain consists of the framework regions (FR1, FR2, FR3, and FR4) and complementarity-determining regions (CDR1, CDR2, and CDR3). To determine the individual gene segments employed by VDJ and VJ rearrangements and the number of nucleotide mutations and amino acid replacements, the variable domain sequences were aligned with germline gene segments using the international ImMunoGeneTics (IMGT) alignment tool.

Abbreviations: Vh, variable gene segment of the heavy chain variable domain; Dh, diversity gene segment of the heavy chain variable domain; Jh, joining gene segment of the heavy chain variable domain; rf, reading frame; V_H, heavy chain variable domain; Mut, mutation number; Vl, variable gene segment of the light chain variable domain; Jl, joining gene segment of the light chain variable domain; V_L, light chain variable domain.

Supplementary Table 2 | X-ray data collection and refinement statistics of Fab 38-1-10A

**Data collection**	
wavelength (Å)	0.9763
Number of crystals	9
Space group	$P\ 2_1\ 2_1\ 2$
Cell dimensions	
a, b, c (Å)	292.4, 87.1, 177.0
α, β, γ (°)	90, 90, 90
Resolution (Å)	67.5-2.70 (2.75-2.70)*
No. reflections	124934 (6090)
CC 1/2	1.0 (0.6)
I / σI	16.4 (1.1)
Rmerge	0.37 (-)
Completeness (%)	100 (99.4)
Redundancy	53.8 (8.1)
**Refinement**	
No. reflections	118703/6151
Rwork / Rfree	0.190/0.240
No. molecules/asymmetric unit	9
No. atoms	
Protein	29206
Water	483
B-factor	
Protein	67
Water	49
R.m.s. deviations	
Bond lengths (Å)	0.006
Bond angles (°)	1.0
NCS related molecules (Å)	0.7
Ramachandran plot	
Favored (%)	93.8
Allowed (%)	5.1
Outliers (%)	1.1

*Numbers in the brackets are for the highest resolutions shell.

Supplementary Table 3 | Cryo-EM data collection and refinement statistics of EV71/38-1-10A and EV71/38-3-11A complexes

	**EV71/38-1-10A**	**EV71/38-3-11A**
**Data collection and reconstruction**		
Voltage (kV)	300	300
Frames	40	30
Dose rate (e^{-}/ Å ^{2}/ s)	4	5
Total dose (e^{-}/ Å ^{2})	30	41
Pixel size (Å)	1.35	1.05
Defocus (μm)	0.5-2.5	1-3.4
Movies	324	2084
Particles	10074	14430
Map resolution (Å)	2.7	2.8
Map sharpening B-factor (Å^{2})	-90.0	-154.4
**Model refinement**		
Model-to-map fit, CC_mask	0.87	0.86
R.m.s.d., bonds (Å)	0.004	0.005
R.m.s.d., angles (°)	0.6	0.7
All-atom Clash score	9.5	8.7
Rotamer outliers (%)	3.8	3.5
Ramachandran plot		
Favored (%)	96.0	94.8
Allowed (%)	4.0	5.2
Outliers (%)	0	0

Supplementary Table 4 | The complete VP2-VP3-VP1 sequences of EV71.

Virus	Type	Year	Region	Sequence
BrCr	A	1970	USA	SPSAEACGYSDRVAQLTIGNSTITTQEAANIIVGYGEWPSYCSDNDATAVDKPTRP<br>DVSVNRFYTLDTKLWEKSSKGWYWKFPDVLTETGVFGQNAQFHYLYRSGFCIH<br>VQCNASKFHQGALLVAVLPEYVIGTVAGGTGTENSHPPYKQTQPGADGFELQHP<br>YVLDAGIPISQLTVCPHQWINLRTNNCATIIVPYMNTLPFDSALNHCNFGLLVVPI<br>SPLDFDQGATPVIPITITLAPMCSEFAGLRQAVTQGFPTELKPGTNQFLTTDDGVS<br>APILPNFHPTPCIHIPGEVRNLLELCQVETILEVNNVPTNATSLMERLRFPVSAQA<br>GKGELCAVFRADPGRDGPWQSTMLGQLCGYYTQWSGSLEVTFMFTGSFMATGK<br>MLIAYTPPGGPLPKDRATAMLGTHVIWDFGLQSSVTLVIPWISNTHYRAHARDG<br>VFDYYTTGLVSIWYQTNYVVPIGAPNTAYIIALAAAQKNFTMKLCKDTSDILQTA<br>TIQGDRVADVIESSIGDSVSKALTQALPAPTGQNTQVSSHRLDTGKVPALQAAEIG<br>ASSNASDESMIETRCVLNSHSTAETTLDSFFSRAGLVGEIDLPLKGTTNPNGYAN<br>WDIDITGHAQMRRKVELFTYMRFDAEFTFVACTPTGEVVPQLLQYMFVPPGAPK<br>PDSRDSLAWQTATNPSVFVKLSDPPAQVSVPFMSPASAYQWFYDGYPTFGEHK<br>QEKDLEYGACPNNMMGTFSVRTVGSSKSKYPLVIRIYMRMKHVRAWIPRPMRN<br>QNYLFKSNPNYAGNSIKPTGTSRTAITTL
98-4215	C1	1998	Taiwan	SPSAEACGYSDRVAQLTIGNSTITTLEAANIIVGYGEWPSYCSDSDATAVDKPTRP<br>DVSVNRFYTLDTKLWEKSSKGWYWKFPDVLTETGVFGQNAQFHYLYRSGFCIH<br>VQCNASKFHQGALLVAVLPEYVIGTVAGGTGTEDSHPPYKQTQPGADGFELQHP<br>YVLDAGIPISQLTVCPHQWINLRTNNCATIIVPYINALPFDSALNHCNFGLLVVHIS<br>PLDFDQGATPVIPITITLAPMCSEFAGLRQAVTQGFPTELKPGTNQFLTTDDGVS<br>APILPNFHPTPCIHIPGEVRNLLELCQVETILEVNNVPTNATSLMERLRFPVSAQA<br>GKGELCAVFRADPGRNGPWQSTLLGQLCGYYTQWSGSLEVTFMFTGSFMATGK<br>MLIAYTPPGGPLPKDRATAMLGTHVIWDFGLQSSVTLVIPWISNTHYRAHARDG<br>VFDYYTTGLVSIWYQTNYVVPIGAPNTAYIIALAAAQKNFTMKLCKDASDILQTG<br>TIQGDRVADVIESSIGDSVSRALTQALPAPTGQNTQVSSHRLDTGKVPALQAAEIG<br>ASSNASDESMIETRCVLNSHSTAETTLDSFFSRAGLVGEIDLPLEGTTNPNGYAN<br>WDIDITGYAQMRRKVELFTYMRFDAEFTFVACTPTGQVVPQLLQYMFVPPGAPK<br>PDSRESLAWQTATNPSVFVKLTDPPAQVSVPFMSPASAYQWFYDGYPTFGEHK<br>QEKDLEYGACPNNMMGTFSVRTVGTSKSKYPLVIRIYMRMKHVRAWVPRPMRN<br>QNYLFKANPNYAGNSIKPTGASRTAITTF
99-1691	C2	1999	Taiwan	SPSAEACGYSDRVAQLTIGNSTITTQEAANIIVGYGEWPSYCSDSDATAVDKPTRP<br>DVSVNRFYTLDTKLWEKSSKGWYWKFPDVLTETGVFGQNAQFHYLYRSGFCIH<br>VQCNASKFHQGALLVAVLPEYVIGTVAGGTGTEDSHPPYKQTQPGADGFELQHP<br>YVLDAGIPISQLTVCPHQWINLRTNNCATIIVPYINALPFDSALNHCNFGLLVVPIS<br>PLDYDQGATPVIPITITLAPMCSEFAGLRQAVTQGFPTELKPGTNQFLTTDDGVS<br>APILPNFHPTPCIHIPGEVRNLLELCQVETILEVNNVPTNAASLMERLRFPVSAQA<br>GKGELCAVFRADPGRSGPWQSTLLGQLCGYYTQWSGSLEVTFMFTGSFMATGK<br>MLIAYTPPGGPLPKDRATAMLGTHVIWDFGLQSSVTLVIPWISNTHYRAHARDG<br>VFDYYTTGLVSIWYQTNYVVPIGAPNTAYIIALAAAQKNFTMKLCKDASDILQTG<br>TIQGDRVADVIESSIGDSVSRALTRALPAPTGQDTQVSSHRLDTGKVPALQAAEIG<br>ASSNASDESMIETRCVLNSHSTAETTLDSFFSRAGLVGEIDLPLEGTTNPNGYAN<br>WDIDITGYAQMRRKVELFTYMRFDAEFTFVACTPTGEVVPQLLQYMFVPPGAPK<br>PDSRESLAWQTATNPSVFVKLSDPPAQVSVPFMSPASAYQWFYDGYPTFGEHKQ<br>EKDLEYGACPNNMMGTFSVRTVGTSKSKYPLVIRIYMRMKHVRAWIPRPMRNQ<br>NYLFKANPNYAGNSIKPTGASRTAITTL
16-3443	C4	2016	Taiwan	SPSAEACGYSDRVAQLTIGNSTITTQEAANIIVGYGEWPSYCSDSDATAVDKPTRP<br>DVSVNRFYTLDTKLWEKSSKGWYWKFPDVLTETGVFGQNAQFHYLYRSGFCIH<br>VQCNASKFHQGALLVAVLPEYVIGTVAGGTGTEDTHPPYKQTQPGADGFELQHP<br>YVLDAGIPISQLTVCPHQWINLRTNNCATIIVPYINALPFDSALNHCNFGLLVVPV<br>SPLDYDQGATPVIPITITLAPMCSEFAGLRQAVTQGFPTEPKPGTNQFLTTDDGVS<br>APILPNFHPTPCIHIPGEVRNLLELCQVETILEVNNVPTNATSLMERLRFPVSAQA<br>GKGELCAVFRADPGRNGPWQSTLLGQLCGYYTQWSGSLEVTFMFTGSFMATGK<br>MLIAYTPPGGPLPKDRATAMLGTHVIWDFGLQSSVTLVIPWISNTHYRAHARDG<br>VFDYYTTGLVSIWYQTNYVVPIGAPNTAYIIALAAAQKNFTMKLCKDASDILQTG<br>TIQGDRVADVIESSIGDSVSRALTHALPAPTGQNTQVSSHRLDTGKVPALQAAEIG<br>ASSNASDESMIETRCVLNSHSTAETTLDSFFSRAGLVGEIDLPLEGTTNPNGYAN<br>WDIDITGYAQMRKKVELFTYMRFDAEFTFVACTPTGQVVPQLLQYMFVPPGAPK<br>PDSRESLAWQTATNPSVFVKLSDPPAQVSVPFMSPASAYQWFYDGYPTFGEHKQ<br>EKDLEYGACPNNMMGTFSVRTVGTSKSKYPLVVRIYMRMKHVRAWIPRPMRNQ<br>NYLFKANPNYAGNTIKPTGASRTSITTL
11-96023	C4	2011	Taiwan	SPSAEACGYSDRVAQLTIGNSTITTQEAANIIVGYGEWPSYCSDSDATAVDKPTRP<br>DVSVNRFYTLDTKLWEKSSKGWYWKFPDVLTETGVFGQNAQFHYLYRSGFCIH<br>VQCNASKFHQGALLVAVLPEYVIGTVAGGTGTEDTHPPYKQTQPGADGFELQHP<br>YVLDAGIPISQLTVCPHQWINLRTNNCATIIVPYINALPFDSALNHCNFGLLVVPIS<br>PLDYDQGATPVIPITITLAPMCSEFAGLRQAVTQGFPTELKPGTNQFLTTDDGVS<br>APILPNFHPTPCIHIPGEVRNLLELCQVETILEVNNVPTNATSLMERLRFPVSAQA<br>GKGELCAVFRADPGRNGPWQSTLLGQLCGYYTQWSGSLEVTFMFTGSFMATGK<br>MLIAYTPPGGPLPKDRATAMLGTHVIWDFGLQSSVTLVIPWISNTHYRAHARDG<br>VFDYYTTGLVSIWYQTNYVVPIGAPNTAYIIALAAAQKNFTMKLCKDASDILQTG<br>TIQGDRVADVIESSIGDSVSRALTHALPAPTGQNTQVSSHRLDTGKVPALQAAEIG<br>ASSNASDESMIETRCVLNSHSTAETTLDSFFSRAGLVGEIDLPLEGTTNPNGYAN

				WDIDITGYAQMRRKVELFTYMRFDAEFTFVACTPTGQVVPQLLQYMFVPPGAPK<br>PDSRESLAWQTATNPSVFVKLSDPPAQVSVPFMSPASAYQWFYDGYPTFGEHKQ<br>EKDLEYGACPNNMMGTFSVRTVGTSKSKYPLVVRIYMRMKHVRAWIPRPMRNQ<br>NYLFKANPNYAGNSIKPTGASRTAITTL
07-72043	C5	2007	Taiwan	SPSAEACGYSDRVAQLTIGNSTITTQEAANIIVGYGEWPSYCSDSDATAVDKPTRP<br>DVSVNRFYTLDTKLWEKSSKGWYWKFPDVLTETGVFGQNAQFHYLYRSGFCIH<br>VQCNASKFHQGALLVAVLPEYVIGTVAGGTGTEDSHPPYKQTQPGAEGFELQHP<br>YVLDAGIPISQLTVCPHQWINLRTNNCATIIVPYINACPFDFALNHCNFGLLVVPIS<br>PLDFDQGATPVIPITITLAPMCSEFAGLRQAVTQGFPTELKPGTNQFLTTDDGVS<br>APILPNFHPTPCIHIPGEVRNLLELCQVETILEVNNVPTNATSLMERLRFPVSAQA<br>GKGELCAVFRADPGRSGPWQSTLLGQLCGYYTQWSGSLEVTFMFTGSFMATGK<br>MLIAYTPPGGPLPKDRATAMLGTHVIWDFGLQSSVTLVIPWISNTHYRAHARDG<br>VFDYYTTGLVSIWYQTNYVVPIGAPNTAYIIALAAAQKNFTMKLCKDASDILQTG<br>TIQGDRVADVIESSIGDSVSRALTQALPAPTGQNTQVSSHRLDTGKVPALQAAEIG<br>ASSNASDESMIETRCVLNSHSTAETTLDSFFSRAGLVGEIDLPLEGTTNPNGYAN<br>WDIDITGYAQMRRKVELFTYMRFDAEFTFVACTPTGEVVPQLLQYMFVPPGAPK<br>PDSRESLAWQTATNPSVFVKLSDPPAQVSVPFMSPASAYQWFYDGYPTFGEHKQ<br>EKDLEYGACPNNMMGTFSVRTVGTSKSKYPLVIRIYMRMKHVRAWVPRPMRNQ<br>NYLFKANPNYAGNSIKPTGASRTAITTL
00-2278	B4	2000	Taiwan	SPSAEACGYSDRVAQLTIGNSTITTQEAANIIVGYGEWPSYCSDDDATAVDKPTRP<br>DVSVNRFYTLDTKLWEKSSKGWYWKFPDVLTETGVFGQNAQFHYLYRSGFCIH<br>VQCNASKFHQGALLVAILPEYVIGTVAGGTGTEDSHPPYKQTQPGADGFELQHPY<br>VLDAGIPISQLTVCPHQWINLRTNNCATIIVPYMNTLPFDSALNHCNFGLLVVPIS<br>PLDFDQGATPVIPITITLAPMCSEFAGLRQAVTQGFPTEPKPGTNQFLTTDDGVS<br>APILPNFHPTPCIHIPGEVRNLLELCQVETILEVNNVPTNATSLMERLRFPVSAQA<br>GKGELCAVFRADPGRDGPWQSTMLGQLCGYYTQWSGSLEVTFMFTGSFMATGK<br>MLIAYTPPGGPLPKDRATAMLGTHVIWDFGLQSSVTLVIPWISNTHYRAHARDG<br>VFDYYTTGLVSIWYQTNYVVPIGAPNTAYIIALAAAQKNFTMKLCKDTSHILQTA<br>SIQGDRVADVIESSIGDSVSRALTQALPAPTGQNTQVSSHRLDTGEVPALQAAEIG<br>ASSNTSDESMIETRCVLNSHSTAETTLDSFFSRAGLVGEIDLPLEGTTNPNGYAN<br>WDIDITGYAQMRRKVELFTYMRFDAEFTFVACTPTGGVVPQLLQYMFVPPGAPK<br>PESRESLAWQTATNPSVFVKLTDPPAQVSVPFMSPASAYQWFYDGYPTFGEHKQ<br>EKDLEYGACPNNMMGTFSVRTVGSSKSKYPLVVRIYMRMKHVRAWIPRPMRNQ<br>NYLFKANPNYAGNSIKPTGTSRTAITTL
12-96015	B5	2012	Taiwan	SPSAEACGYSDRVAQLTIGNSTITTQEAANIIVGYGEWPSYCSDDDATAVDKPTRP<br>DVSVNRFYTLDTKLWEKSSKGWYWKFPDVLTETGVFGQNAQFHYLYRSGFCIH<br>VQCNASKFHQGALLVAILPEYVIGTVAGGTGTEDSHPPYKQTQPGADGFELQHPY<br>VLDAGIPISQLTICPHQWINLRTNNCATIIVPYMNTLPFDSALNHCNFGLLVVPISP<br>LDFDQGATPVIPITITLAPMCSEFAGLRQAVTQGFPTEPKPGTNQFLTTDDGVSA<br>PILPNFHPTPCIHIPGEVRNLLELCQVETILEVNNVPTNATSLMERLRFPVSAQAG<br>KGELCAVFRADPGRDGPWQSTMLGQLCGYYTQWSGSLEVTFMFTGSFMATGK<br>MLIAYTPPGGPLPKDRATAMLGTHVIWDFGLQSSVTLVIPWISNTHYRAHARDG<br>VFDYYTTGLVSFWYQTNYVVPIGAPNTAYIIALAAAQKNFTMKLCKDTSHILQTA<br>SIQGDRVADVIESSIGDSVSRALTQALPAPTGQNTQVSSHRLDTGEVPALQAAEIG<br>ASSNTSDESMIETRCVLNSHSTAETTLDSFFSRAGLVGEIDLPLEGTTNPNGYAN<br>WDIDITGYAQMRRKVELFTYMRFDAEFTFVACTPTGQVVPQLLQYMFVPPGAPK<br>PDSRESLAWQTATNPSVFVKLTDPPAQVSVPFMSPASAYQWFYDGYPTFGEHK<br>QEKDLEYGACPNNMMGTFSVRTVGSSKSKYPLVVRIYMRMKHVRAWIPRPMRN<br>QNYLFKANPNYAGNSIKPTGTSRTAITTL
TW-1745	B5	2008	Taiwan	SPSAEACGYSDRVAQLTIGNSTITTQEAANIIVGYGEWPSYCSDDDATAVDKPTRP<br>DVSVNRFYTLDTKLWEKSSKGWYWKFPDVLTETGVFGQNAQFHYLYRSGFCIH<br>VQCNASKFHQGALLVAILPEYVIGTVAGGTGTEDSHPPYKQTQPGADGFELQHPY<br>VLDAGIPISQLTICPHQWINLRTNNCATIIVPYMNTLPFDSALNHCNFGLLVVPISP<br>LDFDQGATPVIPITITLAPMCSEFAGLRQAVTQGFPTEPKPGTNQFLTTDDGVSA<br>PILPNFHPTPCIHIPGEVRNLLELCQVETILEVNNVPTNATSLMERLRFPVSAQAG<br>KGELCAVFRADPGRDGPWQSTMLGQLCGYYTQWSGSLEVTFMFTGSFMATGK<br>MLIAYTPPGGPLPKDRATAMLGTHVIWDFGLQSSVTLVIPWISNTHYRAHARDG<br>VFDYYTTGLVSIWYQTNYVVPIGAPNTAYIIALAAAQKNFTMKLCKDTSHILQTA<br>SIQGDRVADVIESSIGDSVSRALTQALPAPTGQNTQVSSHRLDTGEVPALQAAEIG<br>ASSNTSDESMIETRCVLNSHSTAETTLDSFFSRAGLVGEIDLPLKGTTNPNGYAN<br>WDIDITGYAQMRRKVELFTYMRFDAEFTFVACTPTGEVVPQLLQYMFVPPGAPK<br>PDSRESLAWQTATNPSVFVKLTDPPAQVSVPFMSPASAYQWFYDGYPTFGEHK<br>QEKDLEYGACPNNMMGTFSVRTVGSSKSKYPLVVRIYMRMKHVRAWIPRPMRN<br>QNYLFKANPNYAGNSIKPTGTSRTAITTL
C1009	B5	2015	Taiwan	SPSAEACGYSDRVAQLTIGNSTITTQEAANIIVGYGEWPSYCSDDDATAVDKPTRP<br>DVSVNRFYTLDTKLWEKSSKGWYWKFPDVLTETGVFGQNAQFHYLYRSGFCIH<br>VQCNASKFHQGALLVAILPEYVIGTVAGGTGTEDSHPPYKQTQPGADGFELQHPY<br>VLDAGIPISQLTICPHQWINLRTNNCATIIVPYMNTLPFDSALNHCNFGLLVVPISP<br>LDFDQGATPVIPITITLAPMCSEFAGLRQAVTQGFPTEPKPGTNQFLTTDDGVSA<br>PILPNFHPTPCIHIPGEVRNLLELCQVETILEVNNVPTNATSLMERLRFPVSAQAG<br>KGELCAVFRADPGRDGPWQSTMLGQLCGYYTQWSGSLEVTFMFTGSFMATGK<br>MLIVYTPPGGPLPKDRATAMLGTHVIWDFGLQSSVTLVIPWISNAHYRAHARDG<br>VFDYYTTGLISIWYQTNYVVPIGAPNTAYIIALAAAQKNFTMKLCKDTSHILQTAS

	IQGDRVADVIESSIGDSVSRALTRALPAPTGQNTQVSSHRLDTGEVPALQAAEIGA<br>SSNTSDESMIETRCVLNSHSTAETTLDSFFSRAGLVGEIDLPLEGTTNPNGYANW<br>DIDITGYAQMRRKVELFTYMRFDAEFTFVACTPTGQVVPQLLQYMFVPPGAPKP<br>DSRESLAWQTATNPSVFVKLTDPPAQVSVPFMSPASAYQWFYDGYPTFGEHKQ<br>EKDLEYGACPNNMMGTFSVRTVGSSKSKYPLVIRIYMRMKHVRAWIPRPMRNQ<br>NYLFKANPNYAGNSIKPTGTSRTAITTL

References

1 Arthur Huang, K.-Y. *et al.* Epitope-associated and specificity-focused features of EV71-neutralizing antibody repertoires from plasmablasts of infected children. *Nature Communications* **8**, 762, doi:10.1038/s41467-017-00736-9 (2017).

2 Huang, K. A. *et al.* Structure-function analysis of neutralizing antibodies to H7N9 influenza from naturally infected humans. *Nat Microbiol* **4**, 306-315, doi:10.1038/s41564-018-0303-7 (2019).
