# Supplementary material for: Structural and functional analysis of protective antibodies targeting the threefold plateau of enterovirus 71
Source: Nat Commun. 2020 Oct 16;11:5253. doi: 10.1038/s41467-020-19013-3 (PMC7567869; doi:10.1038/s41467-020-19013-3)
Supplement: Supplementary file 3 — Reporting Summary [file 41467_2020_19013_MOESM3_ESM.pdf]

nature research

Corresponding author(s): K-Y.A.Huang, D.I.Stuart

Last updated by author(s): Aug 22, 2020

Reporting Summary

Nature Research wishes to improve the reproducibility of the work that we publish. This form provides structure for consistency and transparency in reporting. For further information on Nature Research policies, see our Editorial Policies and the Editorial Policy Checklist.

Statistics

For all statistical analyses, confirm that the following items are present in the figure legend, table legend, main text, or Methods section.

n/a	Confirmed	
☐	☒	The exact sample size (n) for each experimental group/condition, given as a discrete number and unit of measurement
☐	☒	A statement on whether measurements were taken from distinct samples or whether the same sample was measured repeatedly
☐	☒	The statistical test(s) used AND whether they are one- or two-sided<br>*Only common tests should be described solely by name; describe more complex techniques in the Methods section.*
☐	☒	A description of all covariates tested
☒	☐	A description of any assumptions or corrections, such as tests of normality and adjustment for multiple comparisons
☐	☒	A full description of the statistical parameters including central tendency (e.g. means) or other basic estimates (e.g. regression coefficient) AND variation (e.g. standard deviation) or associated estimates of uncertainty (e.g. confidence intervals)
☐	☒	For null hypothesis testing, the test statistic (e.g. F, t, r) with confidence intervals, effect sizes, degrees of freedom and P value noted<br>*Give P values as exact values whenever suitable.*
☒	☐	For Bayesian analysis, information on the choice of priors and Markov chain Monte Carlo settings
☒	☐	For hierarchical and complex designs, identification of the appropriate level for tests and full reporting of outcomes
☒	☐	Estimates of effect sizes (e.g. Cohen's d, Pearson's r), indicating how they were calculated

Our web collection on statistics for biologists contains articles on many of the points above.

Software and code

Policy information about availability of computer code

Data collection	GDA, SerialEM, EPU
Data analysis	Xia2-dials, Molrep 11.7.02, REFMAC 5.8.0258, CCP4 7.0.078, COOT 0.8.9.2, PHENIX 1.18.1-3865, SHP, PISA 1.48, PyMOL 2.3.2, CHIMERA 1.13, Rivem, MotionCorr2, CTFFIND4, ETHAN, WARP, Relion 3.0. GraphPadPrism8. Graphs were also presented by Microsoft Excel for Mac 2011 version 14.7.7. To determine the individual gene segments employed by VDJ and VJ rearrangements and the number of nucleotide mutations and amino acid replacements, the variable domain sequences were aligned with germline gene segments using the international ImMunoGeneTics (IMGT) alignment tool (http://www.imgt.org/IMGT_vquest/vquest).

For manuscripts utilizing custom algorithms or software that are central to the research but not yet described in published literature, software must be made available to editors and reviewers. We strongly encourage code deposition in a community repository (e.g. GitHub). See the Nature Research guidelines for submitting code & software for further information.

Data

Policy information about availability of data

All manuscripts must include a data availability statement. This statement should provide the following information, where applicable:

- Accession codes, unique identifiers, or web links for publicly available datasets
- A list of figures that have associated raw data
- A description of any restrictions on data availability

All the dataSource data are provided with this paper. supporting the findings of this study are available within the paper and extended data files. Requests for antibody material should be addressed to Kuan-Ying A. Huang. The data that support the findings of this study are available from the corresponding authors upon request. The coordinates for the crystal structure of Fab 38-1-10A (along with the structure factors), the EV71/Fab 38-1-10A complex and the EV71/Fab 38-3-11A complex are deposited with PDB codes 6Z3K, 6Z3Q, 6Z3P respectively. The EM reconstructions for the Fab 38-1-10A and Fab 38-3-11A complexes are deposited

with EMDB, codes EM-11062 and EM-11061. Source data are provided with this paper.

Field-specific reporting

Please select the one below that is the best fit for your research. If you are not sure, read the appropriate sections before making your selection.

☒ Life sciences ☐ Behavioural & social sciences ☐ Ecological, evolutionary & environmental sciences

For a reference copy of the document with all sections, see nature.com/documents/nr-reporting-summary-flat.pdf

Life sciences study design

All studies must disclose on these points even when the disclosure is negative.

Sample size	For animal studies, no initial power analysis was performed to predetermine sample size. The sample size was chosen to provide a minimum of n=4 for each group in the mice challenge study, which is sufficient given the excellent technical reproducibility and is also based on the hSCARB2 mice resources that were available and the general practice in the EV71 field.<br><br>For structural analysis, 360 degrees of data was collected from each crystal. For the cryo-EM of the EV71_Fab-38-1-10A complex 10,074 particles were used, for the EV71_Fab-38-3-11A complex 14,430 particles were used.
Data exclusions	No animal data were excluded from the analyses.<br><br>For Cryo-EM poor CTF fits were discarded. No other data were excluded.
Replication	For animal studies, the findings of the studies were reproduced across the experiments using different experimental set-up (control and experimental groups, prophylactic and therapeutic experiments). All attempts at replication were successful.<br><br>For structural analysis, data were collected from more than one crystal/grid.
Randomization	For animal studies, all mice were assigned randomly to control and experimental groups.<br><br>For the cryo-EM, particles were automatically picked using ETHAN or WARP. For in vivo study, mice were allocated randomly into each treatment group.
Blinding	No blinding was conducted for animal studies. During the animal study, only hSCARB2-transgenic mice were used and all mice were coded. However, we only have one well-trained and competent technician that is responsible for the EV71 challenge mice study, the technician would know the details and experimental set-up of the animal study.<br><br>For the cryo-EM processing, Relion performs unbiased 2D and 3D classification.

Reporting for specific materials, systems and methods

We require information from authors about some types of materials, experimental systems and methods used in many studies. Here, indicate whether each material, system or method listed is relevant to your study. If you are not sure if a list item applies to your research, read the appropriate section before selecting a response.

Materials & experimental systems

n/a	Involved in the study
☐	☒ Antibodies
☐	☒ Eukaryotic cell lines
☒	☐ Palaeontology and archaeology
☐	☒ Animals and other organisms
☐	☒ Human research participants
☒	☐ Clinical data
☒	☐ Dual use research of concern

Methods

n/a	Involved in the study
☒	☐ ChIP-seq
☐	☒ Flow cytometry
☒	☐ MRI-based neuroimaging

Antibodies

Antibodies used	For identification of human plasmablasts for production of monoclonal antibodies, Pacific Blue Mouse anti-Human CD3 (clone UCHT1, catalog number 558117, BD)(5 μg/ml), FITC Mouse anti-Human CD19 (clone HIB19, catalog number 555412, BD)(1:10 dilution in a 100 μl experimental sample), PE-Cy7 Mouse anti-Human CD27 (clone M-T271, catalog number 560609, BD)(1:20 dilution in a 100 μl experimental sample), APC-H7 Mouse anti-Human CD20 (clone L27, catalog number 641396, BD)(5 μg/ml), PE-Cy5 Mouse anti-Human CD38 (clone HIT2, catalog number 555461, BD)(1:10 dilution in a 100 μl experimental sample) were used.

	For characterization of human antibodies:<br>Primary antibody:<br># Anti-influenza human IgG antibody 4A-14, 10 μg/mL (an anti-influenza H7 monoclonal antibody, published in the reference 15 Huang, K. A. et al. Structure-function analysis of neutralizing antibodies to H7N9 influenza from naturally infected humans. Nat. Microbiol. 4, 306-315 (2019))<br># Mouse anti-EV71 antibody (clone 422-8D-4C-4D, 1:1,000 dilution, Sigma-Aldrich)<br><br>Secondary antibody:<br># Fluorescein isothiocyanate-conjugated Goat anti-human IgG secondary antibodies, 2.5 μg/mL (Thermo Fisher Scientific)<br># Horseradish peroxidase-conjugated Rabbit anti-human IgG secondary antibody, 0.25 μg/mL (Rockland Immunochemicals)
Validation	Pacific Blue Mouse anti-Human CD3 (clone UCHT1, catalog number 558117) is purchased from BD. This antibody has been validated for use in flow cytometry.<br>https://www.bdbiosciences.com/us/applications/research/t-cell-immunology/th-1-cells/surface-markers/human/pacific-blue-mouse-anti-human-cd3-ucht1-also-known-as-ucht-1-ucht-1/p/558117<br><br>FITC Mouse anti-Human CD19 (clone HIB19, catalog number 555412) is purchased from BD. This antibody has been validated for use in flow cytometry.<br>https://www.bdbiosciences.com/eu/p/555412<br><br>PE-Cy7 Mouse anti-Human CD27 (clone M-T271, catalog number 560609) is purchased from BD. This antibody has been validated for use in flow cytometry.<br>https://www.bdbiosciences.com/us/p/560609<br><br>APC-H7 Mouse anti-Human CD20 (clone L27, catalog number 641396) is purchased from BD. This antibody has been validated for use in flow cytometry.<br>https://www.bdbiosciences.com/us/applications/research/stem-cell-research/hematopoietic-stem-cell-markers/human/negative-markers/apc-h7-mouse-anti-human-cd20-l27/p/641396<br><br>PE-Cy5 Mouse anti-Human CD38 (clone HIT2, catalog number 555461) is purchased from BD. This antibody has been validated for use in flow cytometry.<br>https://www.bdbiosciences.com/us/applications/research/t-cell-immunology/regulatory-t-cells/surface-markers/human/pe-cy5-mouse-anti-human-cd38-hit2/p/555461<br><br>Anti-influenza human IgG antibody 4A-14 is an anti-influenza H7 monoclonal antibody and its specificity and application has been published in the reference 15 Huang, K. A. et al. Structure-function analysis of neutralizing antibodies to H7N9 influenza from naturally infected humans. Nat. Microbiol. 4, 306-315 (2019). Antibody 4A-14 works well in the flow cytometry and ELISA assay.<br><br>Anti-EV71 antibody (clone 422-8D-4C-4D) is purchased from Sigma-Aldrich (https://www.merckmillipore.com/TW/zh/product/Anti-Enterovirus-71-Antibody-cross-reacts-with-Coxsackie-A16-clone-422-8D-4C-4D,MM_NF-MAB979?ReferrerURL=https%3A%2F%2Fwww.google.com%2F). This antibody has been validated for use in immunofluorescence for the detection of Enterovirus 71.<br><br>All antibodies used were tested with appropriate negative and positive control samples. The information of all antibodies has been provided above and in the manuscript.

Eukaryotic cell lines

Policy information about cell lines

Cell line source(s)	For virus propagation: RD cells (Sigma-Aldrich), For Fab expression: HEK293T cells (ATCC CRL-11268)
Authentication	All cell lines were frequently checked for cellular morphologies, growth rates and functions, but none of cell lines were authenticated.
Mycoplasma contamination	All cell lines were tested for mycoplasma and found to be mycoplasma-negative (MycoAlert Assay, Lonza and A2H 85011441, Sigma-Aldrich).
Commonly misidentified lines<br>(See ICLAC register)	No commonly misidentified cell lines were used

Animals and other organisms

Policy information about studies involving animals; ARRIVE guidelines recommended for reporting animal research

Laboratory animals	Three-week-old specific pathogen-free hSCARB2-transgenic C57BL/6 mice. Both males and females were used for these studies. hSCARB2-transgenic C57BL/6 mice were housed at room temperature 20-23 °C with a relative humidity between 55 and 60% and kept under a 12-hour light:12-hour dark cycle. The detailed informations are specifically described in "Animal studies" section of the Methods.
Wild animals	No wild animals were used in the study.

Field-collected samples	No field-collected samples were used in the study.
Ethics oversight	Animal experiments were performed in accordance with the protocol approved by the Institutional Animal Care and Use Committee in the Chang Gung University, Taiwan. Experiments were carried out in accordance with the 'Guide for the care and use of laboratory animals', the recommendations of the Institute for Laboratory Animal Research, and Association for Assessment and Accreditation of Laboratory Animal Care International standards.

Note that full information on the approval of the study protocol must also be provided in the manuscript.

Human research participants

Policy information about studies involving human research participants

Population characteristics	For human donors, one female child (5-year-old) who was diagnosed with hand, foot and mouth disease, was prospectively enrolled in Chang Gung Memorial Hospital, Taiwan. Acute EV71 infection was diagnosed by a positive laboratory test for EV71 by RT-PCR and viral isolation in the respiratory specimens.
Recruitment	The donor was prospectively and randomly enrolled and signed informed consent was provided. We analyzed the donor's anti-EV71 antibody response in details. There is no potential self selection bias or other biases that may be present and are likely to impact the results.
Ethics oversight	The study protocol and informed consent were approved by the ethics committee at the Chang Gung Medical Foundation, Taiwan. The study and all associated methods were carried out in accordance with the approved protocol and the Declaration of Helsinki and Good Clinical Practice guidelines.

Note that full information on the approval of the study protocol must also be provided in the manuscript.

Flow Cytometry

Plots

Confirm that:

- [x] The axis labels state the marker and fluorochrome used (e.g. CD4-FITC).
- [x] The axis scales are clearly visible. Include numbers along axes only for bottom left plot of group (a 'group' is an analysis of identical markers).
- [x] All plots are contour plots with outliers or pseudocolor plots.
- [x] A numerical value for number of cells or percentage (with statistics) is provided.

Methodology

Sample preparation	Sample preparation listed in Methods.
Instrument	For plasmablast sorting, using BD FACSAria; For binding assay experiment, using BD FACScanto
Software	FlowJo version v7.6.1
Cell population abundance	Plasmablast population accounts for ~ 0.1% of peripheral lymphocytes (~1.5% of peripheral B cells). Single plasmablast is sorted in the study and used to produce human IgG monoclonal antibody. Around 50% of sorted plasmablasts are positive with immunoglobulin cloning.
Gating strategy	For plasmablast sorting, CD3negCD20negCD19posCD27hiCD38hi plasmablasts were gated from lymphocyte subset and sorted as single cells. For binding assay, the FSC/SSC gate for infected RD cells and using FITC (stained with FITC-conjugated anti-human IgG secondary antibody)-positive gating for identification of human anti-EV71 antibody-bound cells.

- [x] Tick this box to confirm that a figure exemplifying the gating strategy is provided in the Supplementary Information.
