# Supplementary material for: Structural and functional analysis of protective antibodies targeting the threefold plateau of enterovirus 71
Source: Nat Commun. 2020 Oct 16;11:5253. doi: 10.1038/s41467-020-19013-3 (PMC7567869; doi:10.1038/s41467-020-19013-3)
Supplement: Supplementary file 2 — Peer Review File [file 41467_2020_19013_MOESM2_ESM.pdf]

Reviewers' Comments:

Reviewer #1:
Remarks to the Author:
GENERAL COMMENTS:

EV71 continues to cause disease outbreaks around the world. It's important to find effective, highly specific preventive and therapeutic interventions (such as neutralizing antibody). The manuscript reports the structural and functional analysis of human protective antibodies targeting the 3-fold plateau of Enterovirus 71. The central findings are that the investigators isolated and characterized a panel of plasmablast-derived monoclonal antibodies from an infected five-year-old child whose antibody response focused on the plateau epitope near the icosahedral 3-fold axis. They obtained 8 of a total of 19 antibodies target this epitope and three of these potently neutralize the virus, then used cryo-EM and mapped the binding region of neutralizing antibodies 38-1-10A and 38-3-11A. The neutralizing epitopes can guide the development of effective vaccines and immunotherapeutics against EV71. Although this is an important viral pathogen and protective antibodies are presented, there are some question should be considered and revised.

SPECIAL COMMENTS:

Previously, the authors showed that the majority of the canyon- and plateau-specific antibodies that they detected recognize capsid residues that are conserved across virus genotypes. However, they found that specificity to the plateau region (16-3-4D and 34-1-6D) was associated with limited neutralization breadth and significantly lower potency (failed to neutralize or only weakly neutralized genotype C viruses). In this paper, they demonstrated that antibodies 38-1-10A, 38-1-2A and 38-3-11A could cross-neutralize all EV71 genotypes. The cryo-electron microscopy structures of the EV71 virion in complex with Fab fragments of the 38-1-10A and 38-3-11A reveal the details of a conserved epitope formed by residues in the BC and HI loops of VP2 and the BC and HI loops of VP3 spanning the region around the 3-fold axis. They should systematically compare and analyze the different engagement among 38-1-10A, 38-3-11A and 34-1-6D, then it can strongly prove that the key amino acid site located at 3-fold axis which play critical role in cross-neutralization.

The authors only showed that the neutralization titer of the Donor C's convalescent serum (Page5, Lines 96-97), how about the acute phase of infection serum, they should test and compare the neutralization titer of the paired sera. In addition, the authors should describe more information about the collected convalescent and acute phase of infection rerum, such as on which day after illness onset (Page 5, Line 96 and 109).

The authors demonstrated that representative plateau-binding neutralizing antibodies 38-1-10A and 38-3-11A both confer effective protection against lethal challenge with EV71 in hSCARB2-transgenic mice. However, they only explore the prophylactic effect of neutralizing antibody 38-1-10A at different doses and treatment hours, how about the 38-3-11A? Because the two antibodies interact with the epitope in quite distinct ways, the authors should systematically compare the in vivo protection of these two antibodies.

It is recommended to show details about the 2D and 3D classifications of the EV71/38-1-10A complex and EV71/38-3-11A complex (Lines 231-236).

In Supplementary Fig. 11 (Lines 262-263), the authors only aligned four genotypes of EV71 to comparing the epitope residues. However, antibodies 38-1-10A, 38-1-2A and 38-3-11A can cross-neutralized all EV71 genotypes including C1, C2 and B5. These genotypes should be added.

In lines 341-342, they cited the wrong reference 24 about the monoclonal antibodies against coxsackievirus A10, it should be Zhu et al., Sci. Adv. 2018; 4 : eaat7459.

Lines 3434-345. The authors states that antibody 16-3-4D binds the virion weakly compared to other neutralizing antibodies studied. Evidence needs to be provided to support this key point of view.

Reviewer #2:
Remarks to the Author:
In this manuscript, Huang et al isolated and characterized 42 plasmablast-derived monoclonal antibodies and 19 of them bound specifically to EV71. The authors then found two representative plateau-binding neutralizing antibodies 38-1-10A and 38-3-11A both confer effective protection against lethal challenge with EV71 in hSCARB2-transgenic mice. The authors also used cry-electron microscopy structured these potent and protective antibodies revealing the details of a conserved epitope formed by residues in the BC and HI loops of VP2 and of VP3 spanning the region around the 3-fold axis. While the authors reasonably provide protective antibodies against EV71 infection, some conclusions of this manuscript are disjointed and other interpretations were not discussed.

Although EV71 vaccines were developed and approved years ago in China, they only cover genotype C virus, and are limited to use in China at this moment. The neutralizing antibody can fill in the gap for children who are suffer EV71 infection with severe symptoms. The findings from this study are novel and interesting that may be applied in clinical trials in the future.

Other points:

1) The authors pointed out that two representative plateau-binding neutralizing antibodies 38-1-10A and 38-3-11A against virus. However, in Figure 1b, Figure 2a and Figure 2c , neutralizing antibody 38-1-2A also showed a very good antiviral property. And in Figure 2c, we can see no matter Pre- or Post -attachment experiments, the EC50 of 38-1-2A is better than 38-1-10A. Why didn’t the authors choose another two antibodies? The authors need provide a better rationale for focusing on 38-1-10A and 38-3-11A antibodies.

2) The authors later tested the protection of these antibodies against EV71 challenge. We could see that all antibodies showed a pre-treat antiviral property while the protection property was nearly abolished after post infection 72h, so how does this antibody could be used in clinical usage? The authors should explained.

3) In figure 6, the authors showed 38-1-10A and 38-3-11A interact with the same epitope of EV71, while Figure 1b also showed that 38-1-2A had the same binding ability with 38-1-10A and 38-3-11A. Whether this antibody has the same binding epitope? In addition, as mentioned above, it is unclear why the authors chose to focus on 38-1-10A and 38-3-11A, especially 38-1-10A.

4) EV71 uses variable receptors in different tissues/cell types. In this study, the authors only examined the antibodies’ neutralization activity in cells with SCARB2 receptor. Their activity in cells with HSPG and PSGL-1 receptors should also be tested.

Reviewed by Ming-Liang He

Reviewer #3:
Remarks to the Author:
This manuscript presents a comprehensive characterization of the antibodies produced by young patient with EV71 induced HFMD. The study includes characterization of the immune response of the patient (demonstrating that a large portion of the serum titer was lost for a virus with a single point mutation in the previously characterized "plateau epitope" of EV71, isolation of monoclonal antibodies from the patient's circulating plasmablasts, characterization of these antibodies abilities to recognize virus, bind capsids, neutralize wt viruses, and neutralize a panel of monoclonal escape mutants against various epitopes. The study goes on to sequence the neutralizing antibodies show that they have a striking similarity to germline antibodies, characterize the reactivity of the antibodies to panels of previously characterized escape mutants, demonstrate that the antibodies confer protection against IP challenge of receptor transgenic mice, and determine the cryoEM structures of two of the antibodies, and characterize the mechanism of neutralization for these two antibodies. The manuscript is a bit of a tour de force, makes a number of significant observations (some surprising), and represents a significant addition to our understanding of this emerging pathogen.

Please excuse the length of this review, which is more a product of some of the questions the manuscript raised as I read it than a critique. Most of the comments simply reflect questions that might help subsequent readers if the authors choose to do so, none of the comments or questions should require significant revision, and certainly none would require any additional experiments. It was a fun read that sets a very high bar for subsequent work in the field.

General observation and questions: In the introduction the authors state that the patient's serum had lost appreciable titer to a various against viruses with a single point mutation, VP3 E81K. What was the source of this mutation? Was it selected by one of the other "plateau epitope" monoclonal antibodies? Was it a virus being excreted by the patient?

They go on to say (and show in the results) that the plateau epitope monoclonal antibodies isolated from this patient had variable domain sequences remarkably similar to germlines suggesting that they are rapidly selected and therefore would be readily elicited by immunization.

They also go on to show that the VP3 E81K mutation confers resistance to all three of the monoclonal antibodies they characterize in the manuscript, and to a number of other antibodies specific for the plateau epitope and suggest that antibodies to this epitope represent a significant fraction of the immune response observed from a number of patients.

And finally show in the structures that the VP3 E81K mutation is not within the footprint of either of the Fabs from either of the two monoclonal antibodies characterized, but rather disrupts a network of interactions that stabilize a loop in VP3 that is part of the footprint of both.

There are several surprising and seemingly contradictory findings from this series of observations.

1) In this reviewer's understanding of the field single point mutations that confer resistance to all of a significant panel of monoclonal antibodies that map to an epitope is rare, and a single mutation that significantly reduces the neutralizing titer in serum even more rare. If this epitope is indeed very important, and this single point mutation is indeed so dominant in allowing virus to escape antibodies to this epitope (even in serum) it would seem that the virus would rapidly be fixed in the circulating virus.

-Unless my recollection is wrong it might be worth noting that a significant loss in serum titer due to a point mutation is unusual.
-How common is the mutation in field isolates of the virus?
Is it also common in virus samples isolated during the course of infection?
-Does this point mutation confer a significant loss of fitness for the virus in the absence of the selecting antibodies.
-The authors might like to comment on the implications of these finding for vaccines and for monoclonal therapy (at the very least they suggest that plateau epitope monoclonal antibodies might be a poor choice unless used in combination with monoclonals that recognize other epitopes.

2) The observation that the mutation is not within the binding footprint of either of the antibodies seems surprising. In the past before high-resolution structures of virus-Fab complexes were available it was widely assumed that point escape mutations were a surrogate map for the binding footprint of an antibody. Now that these high-resolution virus Fab structures are becoming more common, is it unusual that a point mutation outside the binding site work by disrupting the structure of an essential component of the epitope common. If not, it is worth pointing out more directly that his is unusual.
3) The authors ascribe the effect of this mutation as a disruption of a hydrogen bonding network that disrupts the structure of the loop that forms part of the binding site for the antibody. The mutation would also be expected to create a significant change in the electrostatic character of in the vicinity of the binding site. Unless there is evidence that preclude this possibility (e.g. structure of the point mutant, or ionic strength dependence of the binding of the antibodies it might be good to hedge bets and mention both possible mechanisms.

Specific comments:

A) It is tough to decipher exactly what is going on in figure 1b, due to some shorthand used by the authors in the caption. I gather, but am not sure, that the column "binding to EV71" means that the antibody scores positive in both the flow cytometry and the ELISA assay", and that the column "reactive to capsids" means scores positive in immunoprecipitation assays. Is this the case? Presumably the antibodies that "bind to EV71" but are not "reactive with capsids" are the expanded particles. This is a bit confusing because one would expect the ELISA to be displaying either predominantly expanded forms (as it does in many other enteroviruses) in which case the viruses that score positive for both the ELISA and the immunoprecipation would be surprising unless the native structure is partially persevered in direct (as opposed to sandwich format) ELISAs for EV71. The caption and the text could be more clear.
B) The observation that the two plateau binding antibodies bind very differently is a bit surprising, but may be related to previous observations that the "plateau epitope" which was seen in panels of monoclonal antibodies to type 1 poliovirus segregated into two subsites based on partial overlap in the cluster analysis of two groups of antibodies and their selected epitope(Page et al. J Virol. 1988 May;62(5):1781-94.) this might be worth noting
C) The section discussing the mechanism of neutralization seems a bit strange. The authors observe that the antibodies neutralize at low concentrations by blocking binding, but at high concentrations also block subsequent steps. This raises two questions: 1) how many copies of the antibody per virion (assuming that the Kd is such that the ratio of bound per virion is the same as the ratio of Ab added per virion) does it take to block binding. 2) It the concentration of Ab required to neutralize virus by blocking binding is much lower than the concentration required to block subsequent steps what is the relevance of the blocking at subsequent steps?

REVIEWERS COMMENTS ADDRESSED POINT BY POINT:

Reviewer #1 (Remarks to the Author):

GENERAL COMMENTS:

EV71 continues to cause disease outbreaks around the world. It's important to find effective, highly specific preventive and therapeutic interventions (such as neutralizing antibody). The manuscript reports the structural and functional analysis of human protective antibodies targeting the 3-fold plateau of Enterovirus 71. The central findings are that the investigators isolated and characterized a panel of plasmablast-derived monoclonal antibodies from an infected five-year-old child whose antibody response focused on the plateau epitope near the icosahedral 3-fold axis. They obtained 8 of a total of 19 antibodies target this epitope and three of these potently neutralize the virus, then used cryo-EM and mapped the binding region of neutralizing antibodies 38-1-10A and 38-3-11A. The neutralizing epitopes can guide the development of effective vaccines and immunotherapeutics against EV71. Although this is an important viral pathogen and protective antibodies are presented, there are some question should be considered and revised.

SPECIAL COMMENTS:

Previously, the authors showed that the majority of the canyon- and plateau-specific antibodies that they detected recognize capsid residues that are conserved across virus genotypes. However, they found that specificity to the plateau region (16-3-4D and 34-1-6D) was associated with limited neutralization breadth and significantly lower potency (failed to neutralize or only weakly neutralized genotype C viruses). In this paper, they demonstrated that antibodies 38-1-10A, 38-1-2A and 38-3-11A could cross-neutralize all EV71 genotypes. The cryo-electron microscopy structures of the EV71 virion in complex with Fab fragments of the 38-1-10A and 38-3-11A reveal the details of a conserved epitope formed by residues in the BC and HI loops of VP2 and the BC and HI loops of VP3 spanning the region around the 3-fold axis. They should systematically compare and analyze the different engagement among 38-1-10A, 38-3-11A and 34-1-6D, then it can strongly prove that the key amino acid site located at 3-fold axis which play critical role in cross-neutralization.

- **We have revised the manuscript to clarify these aspects. We showed the epitope recognized by 34-1-6D only partially overlaps with that of neutralizing antibody 38-1-10A in the revised manuscript (Supplementary Fig. 5) (page 6). We also showed that the epitopes recognized by other 3-fold plateau-binding antibodies (i.e. 38-1-11A, 38-2-2D, 38-B1-2B, 38-1-7B, 38-B2-9B) overlap at some level with that of 38-1-10 but did not neutralize the virus (Supplementary Fig. 5) (page 6). Furthermore, 38-1-10A and 38-3-11A bind largely overlapping sites with radically different poses exhibited varied neutralizing activities. These results indicate that the interaction pattern between the 3-fold plateau epitope and the binding antibody substantially affect the functional phenotype of antibodies. We are planning to derive structures for 34-1-6D and other 3-fold plateau-binding antibodies, but it is beyond the scope of this paper.**

- **In the present study, we focused on the characterization of neutralizing antibodies derived from donor C and showed the relationship of polyclonal serology and plasmablast-derived antibody repertoire at the functional and structural level. We understand that 3-fold plateau-binding neutralizing antibodies were tested with a small panel of EV71 strains representative of various genotypes in the study and further structure–function relationships of each antibody (i.e. key epitope residues, binding affinity) warrants further**

systemic investigation. We have revised the manuscript to point out this limitation (page 15).

The authors only showed that the neutralization titer of the Donor C's convalescent serum (Page5, Lines 96-97), how about the acute phase of infection serum, they should test and compare the neutralization titer of the paired sera. In addition, the authors should describe more information about the collected convalescent and acute phase of infection rerum, such as on which day after illness onset (Page 5, Line 96 and 109).

- **In the study, donor C was enrolled at day 6 and provided whole blood samples at day 7 and 9 after onset of illness. PBMCs were separated from the day 7 sample and used for the isolation of plasmablasts, but there was no serum collected at that day. We had donor C's serum only from the day 9 sample and its neutralization titers with EV71 were tested and presented in the Figure 1a (pages 5, 33, 34).**

- **We have revised the manuscript and the legend of Figure 1a to provide additional information about those convalescent sera tested in the study (pages 5, 33, 34).**

The authors demonstrated that representative plateau-binding neutralizing antibodies 38-1-10A and 38-3-11A both confer effective protection against lethal challenge with EV71 in hSCARB2-transgenic mice. However, they only explore the prophylactic effect of neutralizing antibody 38-1-10A at different doses and treatment hours, how about the 38-3-11A? Because the two antibodies interact with the epitope in quite distinct ways, the authors should systematically compare the in vivo protection of these two antibodies.

- **In the prophylactic experiment, both 38-1-10A and 38-3-11A were effective against lethal challenge of EV71 in hSCARB2-transgenic mice. Nevertheless, while we designed and conducted therapeutic experiments in the hSCRAB2-transgenic mice, we were still collecting preliminary data of 38-3-11A for its antigenic breadth and functional activity against a panel of EV71. At that time, we had fully characterized antibodies 38-1-10A and learned that this antibody exhibited potent activities against both C4 and B5 viruses. In addition, only a limited number of pups born from each pregnancy for hSCARB2-transgenic mice and were available for the experiment. Therefore, 38-1-10A was further tested in the hSCARB2-transgenic mice for the assessment of therapeutic effects at different doses and treatment timepoints. We agree that antibody 38-3-11A could be a promising candidate for *in vivo* studies considering its neutralizing activity and breadth and therapeutic study may be conducted in the near future.**

It is recommended to show details about the 2D and 3D classifications of the EV71/38-1-10A complex and EV71/38-3-11A complex (Lines 231-236).

- **This has been added**

In Supplementary Fig. 11 (Lines 262-263), the authors only aligned four genotypes of EV71 to comparing the epitope residues. However, antibodies 38-1-10A, 38-1-2A and 38-3-11A can cross-neutralized all EV71 genotypes including C1, C2 and B5. These genotypes should be added.

- **This has been done**

In lines 341-342, they cited the wrong reference 24 about the monoclonal antibodies against coxsackievirus A10, it should be Zhu et al., Sci. Adv. 2018; 4 : eaat7459.

- **We have corrected reference 24 in the revised manuscript (pages 15, 30).**

Lines 3434-345. The authors states that antibody 16-3-4D binds the virion weakly compared to other neutralizing antibodies studied. Evidence needs to be provided to support this key point of view.

- **We have revised the manuscript to clarify the findings about antibody16-3-4D (page 15).**

Reviewer #2 (Remarks to the Author):

In this manuscript, Huang et al isolated and characterized 42 plasmablast-derived monoclonal antibodies and 19 of them bound specifically to EV71. The authors then found two representative plateau-binding neutralizing antibodies 38-1-10A and 38-3-11A both confer effective protection against lethal challenge with EV71 in hSCARB2-transgenic mice. The authors also used cry-electron microscopy structured these potent and protective antibodies revealing the details of a conserved epitope formed by residues in the BC and HI loops of VP2 and of VP3 spanning the region around the 3-fold axis. While the authors reasonably provide protective antibodies against EV71 infection, some conclusions of this manuscript are disjointed and other interpretations were not discussed.

Although EV71 vaccines were developed and approved years ago in China, they only cover genotype C virus, and are limited to use in China at this moment. The neutralizing antibody can fill in the gap for children who are suffer EV71 infection with severe symptoms. The findings from this study are novel and interesting that may be applied in clinical trials in the future.

Other points:

1) The authors pointed out that two representative plateau-binding neutralizing antibodies 38-1-10A and 38-3-11A against virus. However, in Figure 1b, Figure 2a and Figure 2c , neutralizing antibody 38-1-2A also showed a very good antiviral property. And in Figure 2c, we can see no matter Pre- or Post - attachment experiments, the EC50 of 38-1-2A is better than 38-1-10A. Why didn't the authors choose another two antibodies? The authors need provide a better rationale for focusing on 38-1-10A and 38-3-11A antibodies.

- **Antibody 38-1-2A was one of three neutralizing antibodies characterized in detail in the study. Antibody 38-1-2A has identical heavy and light chain variable germline gene segment (Vh4-39*01/Dh3-22*01/Jh5*02 and Vk1-6*01/Jk4*01) and CDR3 (HCDR3 AREITMIAWFDP and KCDR3 LQDYNYLLT) regions with 38-1-10A, indicating both antibodies were derived from clonally related B cells (Supplementary Table 1) (Fig. 2b) (pages 6, 8). Both had comparable neutralizing activities against EV71 98-4215 (C1), 16-3443 (C4), 07-72043 (C5), 00-2278 (B4) and 12-96015 (B5) (Fig. 2a) (page 8). Both had comparable pre- and post-attachment activities against EV71 12-96015 (Fig. 2c). In addition, both were sensitive to substitutions of VP3 E81, VP3 S74 and VP2 E72 of EV71 escape mutants, indicating both recognize substantially overlapping epitopes (Supplementary Fig. 4c, Supplementary Fig. 5) (page 6).**

- **Antibody 38-1-10A was the first neutralizing antibody to be isolated from donor C and fully characterized functionally. Thus 38-1-10A was selected first for the high-resolution structural analysis of EV71-Fab analysis. Later on, neutralizing antibodies 38-1-2A and 38-3-11A were isolated and fully characterized. Sequence analysis revealed that antibodies 38-1-2A and 38-1-10A were clonally related. By contrast, antibody 38-3-11A was encoded by a distinct pair of genes for heavy and light chain variable domains and exhibited stronger potencies against EV71 BrCr (A), 98-4215 (C1), 99-1691 (C2), 16-3443 (C4) and 12-96015 (B5) (Fig. 2a, 2b). We therefore selected 38-3-11A for further structural analysis.**

2) The authors later tested the protection of these antibodies against EV71 challenge. We could see that all antibodies showed a pre-treat antiviral property while the protection property was nearly abolished after post infection 72h, so how does this antibody could be used in clinical usage? The authors should explained.

- **We have further provided the discussion related to the findings of *in vivo* study in the revised manuscript (pages 17, 18).**

- **In the present study, the prophylactic and therapeutic efficacies of plateau-binding neutralizing antibodies were assessed in hSCARB2-transgenic mice. The results of prophylactic experiments indicated that potent monoclonal antibodies might be promising candidates as a prophylactic for high-risk population (i.e. young infants or immunocompromised individuals) exposed to virus (refs 6, 20, 26). The therapeutic experiment showed that 38-1-10A substantially reduced the mortality and loss of body weight when administered within 48 hours of challenge. This successful proof-of-concept utilization of a neutralizing antibody is supportive of the use of monoclonal antibody for the post-exposure treatment of lethal EV71 infection, although the mechanism of time dependent effect of 38-1-10A on protection is not well understood. A multi-antibody cocktail approach that targeting non-overlapping neutralizing epitopes might improve or enhance the protection (ref 6). Nevertheless, the formulation and timepoint of antibody administration require more investigations to provide a definitive therapeutic strategy in future clinical studies.**

3) In figure 6, the authors showed 38-1-10A and 38-3-11A interact with the same epitope of EV71, while Figure 1b also showed that 38-1-2A had the same binding ability with 38-1-10A and 38-3-11A. Whether this antibody has the same binding epitope? In addition, as mentioned above, it is unclear why the authors chose to focus on 38-1-10A and 38-3-11A, especially 38-1-10A.

- **Antibody 38-1-2A has identical heavy and light chain variable germline gene segment and CDR3 regions with 38-1-10A, indicating both antibodies were derived from clonally related B cells (Supplementary Table 1) (Fig. 2b) (pages 6, 8). Both had similar neutralizing potencies and breadth against EV71 (Fig. 2a). The competitive binding experiment showed both were sensitive to substitutions of VP3 E81, VP3 S74 and VP2 E72 in the EV71 mutants, indicating 38-1-2A and 38-3-11A recognize substantially overlapping epitopes (Supplementary Fig. 4c).**

- **We characterized neutralizing potencies and breadth for all three neutralizing antibodies (38-1-10A, 38-1-2A and 38-3-11A) in detail in the study. Antibody 38-1-10A was the first neutralizing antibody to be isolated from donor C and fully characterized functionally. Furthermore, we learnt that 38-1-2A is clonally related to 38-1-10A and both recognize substantially overlapping epitopes. Therefore, we proceed with structural analysis with another potent antibody 38-3-11A.**

- **We have clarified the reasons for not taking 38-1-2A further in the revised manuscript (pages 6, 8, 15).**

4) EV71 uses variable receptors in different tissues/cell types. In this study, the authors only examined the antibodies' neutralization activity in cells with SCARB2 receptor. Their activity in cells with HSPG and PSGL-1 receptors should also be tested.

- **Previous studies demonstrated that human RD cells could express SCARB2 and heparan sulfate (refs 10, 11) and this cell line was used in the neutralization assay in the present study. SCARB2 is one of major cellular receptors for EV71 and the EV71-SACRB2 complex reveals the receptor-binding footprint on the viral capsid (ref 12). The overlaying EV71/38-1-10A and EV71/38-3-11A with EV71/SCARB2 shows severe clashes between the Fabs and SCARB2 receptor, suggesting that neutralization could be by preventing receptor binding**

(Supplementary Fig. 13) (page 17). Heparan sulfate serves as an attachment receptor for EV71 (ref 10). Tan et al showed that lysine residues at 162, 242, and 244 of the EV71 capsid VP1 are responsible for electrostatic interactions with heparan sulfate. These residues are at the 5-fold vertex of the capsid and far away from the 3-fold plateau epitope recognized by 38-1-10A, 38-1-2A and 38-3-11A.

- **RD cells express very little or undetectable levels of PSGL-1 (ref 9). Jurkat cells and mouse L929 cells express high level of PSGL-1 and are most commonly used to study the interaction between EV71 and PSGL-1 (refs 8, 9). However, these cell lines are currently unavailable in our laboratory. Besides, Nishimura et al showed that PSGL-1 binds to positively charged amino acids (VP1 residues K242 and K244) located near the 5-fold axis of the EV71 virion via an electrostatic interaction (refs 8, 9). These conserved lysine residues on the virus surface are responsible for interaction with the PSGL-1 and not within the footprints of neutralizing antibodies 38-1-10A and 38-3-11A based on the structural data.**

- **We agree that there is a lack of direct evidence of the potential interference of our neutralizing antibodies with EV71 binding to heparan sulfate and PSGL-1, especially while a high-resolution structures of EV71-heparan sulfate and EV71-PSGL-1 complex remain undetermined at the moment. Cell-based experiments using certain cell lines and pull-down assays may add indirect information about the role of antibodies in the interference of EV71 with heparan sulfate and PSGL-1, but this is beyond the scope of present study. We focused on a detailed mapping of antibody responses, from single cell sequencing through to high resolution structural analysis, and delineated critical epitopes on the viral capsid. We appreciate this valuable comment about the delineation of neutralizing activity of antibodies in cells with heparan sulfate proteoglycans and PSGL-1 receptors and we have revised the manuscript to point out our limitation (page 17).**

Reviewed by Ming-Liang He

Reviewer #3 (Remarks to the Author):

This manuscript presents a comprehensive characterization of the antibodies produced by young patient with EV71 induced HFMD. The study includes characterization of the immune response of the patient (demonstrating that a large portion of the serum titer was lost for a virus with a single point mutation in the previously characterized “plateau epitope” of EV71, isolation of monoclonal antibodies from the patient’s circulating plasmablasts, characterization of these antibodies abilities to recognize virus, bind capsids, neutralize wt viruses, and neutralize a panel of monoclonal escape mutants against various epitopes. The study goes on to sequence the neutralizing antibodies show that they have a striking similarity to germline antibodies, characterize the reactivity of the antibodies to panels of previously characterized escape mutants, demonstrate that the antibodies confer protection against IP challenge of receptor transgenic mice, and determine the cryoEM structures of two of the antibodies, and characterize the mechanism of neutralization for these two antibodies. The manuscript is a bit of a tour de force, makes a number of significant observations (some surprising), and represents a significant addition to our understanding of this emerging pathogen.

Please excuse the length of this review, which is more a product of some of the questions the manuscript raised as I read it than a critique. Most of the comments simply reflect questions that might help subsequent readers if the authors choose to do so, none of the comments or questions should require significant revision, and certainly none would require any additional experiments. It was a fun read that sets a very high bar for subsequent work in the field.

General observation and questions: In the introduction the authors state that the patient’s serum had lost appreciable titer to a various against viruses with a single point mutation, VP3 E81K. What was the source of this mutation? Was it selected by one of the other “plateau epitope” monoclonal antibodies? Was it a virus being excreted by the patient?

- **The virus (11-96023_VP3 E81K) was an escape variant selected *in vitro* with 3-fold plateau neutralizing monoclonal antibody, identified when we mapped the epitopes for a panel of neutralizing monoclonal antibodies earlier (ref 13). TW-1745_VP3 E81K was generated *in vitro* by introducing the residue substitution into an infectious cDNA clone of EV71 (strain TW-1745, GenBank accession number KT354870.1) via site-directed mutagenesis to further confirm the role of the substitution in antibody binding and neutralization in the previous study (ref 13) (the legend of Fig. 1, page 34) (Methods, pages 5, 19, 22).**

- **The mutants were used to screen post-infection sera to examine the breadth of polyclonal antibodies elicited upon acute EV71 infection in children (ref 13). The result revealed that a small subset of post-infection sera exhibited a significantly reduced neutralization titer with the 3-fold plateau epitope mutant than maternal wild type virus (ref 13). Thus, we produced and characterized plasmablast-derived monoclonal antibodies from an individual (donor C) who had a focused serological response to the 3-fold plateau epitope of EV71 capsid in the present study.**

- **We have further clarified the mutants used in the serological assay in the revised manuscript (the legend of Fig 1a; pages 5, 34)**

- **The 3-fold plateau epitope was conserved in the EV71 strain (C1009) isolated from donor C (Supplementary Table 4). Donor C’ convalescent serum showed a strong neutralization titer (1:1024) to EV71 C1009 which is comparable to the titer to wild type 11-96023 strain (Supplementary Fig. 1).**

They go on to say (and show in the results) that the plateau epitope monoclonal antibodies isolated from this patient had variable domain sequences remarkably similar to germlines suggesting that they are rapidly selected and therefore would be readily elicited by immunization.

They also go on to show that the VP3 E81K mutation confers resistance to all three of the monoclonal antibodies they characterize in the manuscript, and to a number of other antibodies specific for the plateau epitope and suggest that antibodies to this epitope represent a significant fraction of the immune response observed from a number of patients.

And finally show in the structures that the VP3 E81K mutation is not within the footprint of either of the Fabs from either of the two monoclonal antibodies characterized, but rather disrupts a network of interactions that stabilize a loop in VP3 that is part of the footprint of both.

There are several surprising and seemingly contradictory findings from this series of observations.

1) In this reviewer's understanding of the field single point mutations that confer resistance to all of a significant panel of monoclonal antibodies that map to an epitope is rare, and a single mutation that significantly reduces the neutralizing titer in serum even more rare. If this epitope is indeed very important, and this single point mutation is indeed so dominant in allowing virus to escape antibodies to this epitope (even in serum) it would seem that the virus would rapidly be fixed in the circulating virus.

-Unless my recollection is wrong it might be worth noting that a significant loss in serum titer due to a point mutation is unusual.
-How common is the mutation in field isolates of the virus?
Is it also common in virus samples isolated during the course of infection?
-Does this point mutation confer a significant loss of fitness for the virus in the absence of the selecting antibodies.
-The authors might like to comment on the implications of these finding for vaccines and for monoclonal therapy (at the very least they suggest that plateau epitope monoclonal antibodies might be a poor choice unless used in combination with monoclonals that recognize other epitopes.

- **In the previous study, post-infection sera were tested with EV71 11-96023 (genotype C4) escape variants (selected *in vitro* with neutralizing monoclonal antibodies), and 3 of 27 sera tested had a 16-fold reduction in titers with variant harboring single mutation on the viral capsid epitope (VP1 D164V at the canyon epitope, VP1 K162E at the canyon epitope, or VP3 E81K at the 3-fold plateau epitope) (ref 13). We agree that it is not common to detect a focused serological response upon natural EV71 infection among patients that is sensitive to a point mutation on the viral capsid. We have further pointed out this finding in the revised manuscript (ref 13) (page 4). We have clarified the mutants used in the serological assay in the revised manuscript as well (the legend of Fig 1a; pages 5, 34).**

- **Previously, we isolated a broadly reactive neutralizing antibody 16-3-3C that selected single residue mutation at VP1 164. This amino acid change was also noted in genotype B4 circulating viruses and could be linked to the resistance of genotype B4 strains to antibody 16-3-3C (ref 13). This is a typical example of monoclonal antibodies isolated from such individuals selecting for residue changes observed in nature. Nevertheless, the majority of viral capsid epitopes**

recognized by neutralizing monoclonal antibodies derived from EV71-infected individuals at the moment are conserved among genotypes B and C EV71 viruses isolated in the last decade. Indeed the 3-fold plateau epitope recognized by antibodies 38-1-10A, 38-1-2A and 38-3-11A is conserved in the circulating EV71 (Supplementary Fig. 11, Supplementary Table 4). Thus the role of highly focused antibody responses, which we detect in a small subset of EV71 pediatric patients, in driving antigenic change (epitope mutation) in the circulating strains remains unclear and warrants further investigation of the human antibody repertoire and epidemiological surveillance of circulating strains in the future.

- **In the present study, escape mutants from 11-96023 (genotype C4) and 00-2278 (genotype B4) viruses were selected in the presence of neutralizing antibody (38-1-10A, 38-3-11A) *in vitro*. The mutants showed infectivity for RD cell line similar to that of parent strain (~$7.9x10^8$ TCID50/ml for 11-96023 and ~$7.4x10^8$ for 00-2278 mutants). These results suggest that there is no significant loss of fitness for the escape variant in this cell line. We have provided the related information in the revised manuscript (Methods, page 22).**

- **In the present study, we showed the prophylactic and therapeutic efficacies of plateau-binding neutralizing antibodies in hSCARB2-transgenic mice. The results of prophylactic experiments indicated that potent monoclonal antibodies might be promising candidates as a prophylactic for high-risk populations (i.e. young infants or immunocompromised individuals) exposed to virus. 38-1-10A substantially reduced the mortality and loss of body weight when administered within 48 hours of challenge. This is supportive of the use of monoclonal antibody for post-exposure treatment of lethal EV71 infection, although the mechanism of the time dependence of protection by 38-1-10A is not well understood. We agree that a multi-antibody cocktail approach targeting non-overlapping neutralizing epitopes might improve or enhance the protection and this strategy has been used for anti-HIV and anti-Ebola neutralizing monoclonal antibodies *in vivo*. Nevertheless, the logistics require more investigation to provide a definitive therapeutic strategy. We have discussed this in the revised manuscript (pages 17, 18).**

2) The observation that the mutation is not within the binding footprint of either of the antibodies seems surprising. In the past before high-resolution structures of virus-Fab complexes were available it was widely assumed that point escape mutations were a surrogate map for the binding footprint of an antibody. Now that these high-resolution virus Fab structures are becoming more common, is it unusual that a point mutation outside the binding site work by disrupting the structure of an essential component of the epitope common. If not, it is worth pointing out more directly that his is unusual.

- **Thanks for this, yes it remains unusual in our view and we have pointed this out more clearly.**

3) The authors ascribe the effect of this mutation as a disruption of a hydrogen bonding network that disrupts the structure of the loop that forms part of the binding site for the antibody. The mutation would also be expected to create a significant change in the electrostatic character of in the vicinity of the binding site. Unless there is evidence that preclude this possibility (e.g. structure of the point mutant, or ionic strength dependence of the binding of the antibodies it might be good to hedge bets and mention both possible mechanisms.

- **Agreed, we have amended the text.**

Specific comments:

A) It is tough to decipher exactly what is going on in figure 1b, due to some shorthand used by the authors in the caption. I gather, but am not sure, that the column “binding to EV71” means that the antibody scores positive in both the flow cytometry and the ELISA assay”, and that the column “reactive to capsids” means scores positive in immunoprecipitation assays. Is this the case? Presumably the antibodies that “bind to EV71” but are not “reactive with capsids” are the expanded particles. This is a bit confusing because one would expect the ELISA to be displaying either predominantly expanded forms (as it does in many other enteroviruses) in which case the viruses that score positive for both the ELISA and the immunoprecipation would be surprising unless the native structure is partially persevered in direct (as opposed to sandwich format) ELISAs for EV71. The caption and the text could be more clear.

- **In the present study, a total of 19 monoclonal antibodies were specifically reactive to EV71 in the flow cytometry. Eight of the 19 anti-EV71 antibodies reacted to purified native viruses in the direct ELISA and the viral capsid by immunoprecipitation (Fig. 1b, Supplementary Fig. 3). The other 11 antibodies may either be capsid-specific but fail to react with native viruses in the direct ELISA and immunoprecipitation or react to other viral antigens (i.e. internal proteins) expressed in infected cells by flow cytometry. We focused on the characterization of EV71 capsid-reactive antibodies and three neutralizing antibodies in the present study. We have revised Fig. 1b to clarify the characterization of 19 plasmablast-derived EV71-specific monoclonal from donor C in the manuscript (pages 33, 34).**

B) The observation that the two plateau binding antibodies bind very differently is a bit surprising, but may be related to previous observations that the “plateau epitope” which was seen in panels of monoclonal antibodies to type 1 poliovirus segregated into two subsites based on partial overlap in the cluster analysis of two groups of antibodies and their selected epitope(Page et al. J Virol. 1988 May;62(5):1781-94.) this might be worth noting

- **Now noted**

C) The section discussing the mechanism of neutralization seems a bit strange. The authors observe that the antibodies neutralize at low concentrations by blocking binding, but at high concentrations also block subsequent steps. This raises two questions: 1) how many copies of the antibody per virion (assuming that the Kd is such that the ratio of bound per virion is the same as the ratio of Ab added per virion) does it take to block binding. 2) It the concentration of Ab required to neutralize virus by blocking binding is much lower than the concentration required to block subsequent steps what is the relevance of the blocking at subsequent steps?

- **We acknowledge that information on the stoichiometry of antibody-mediated neutralization could be valuable to understand the number of copies of antibody per virion required to block the binding and subsequent post-attachment step, but a limited of laboratory resources are available in the current COVID19 outbreak and this experiment is unfortunately beyond the scope of the present study.**
